# OSeMOSYS-RDM: A reproducible workflow for robust decision making with OSeMOSYS models

**DOI:** 10.1016/j.mex.2026.104102

**Published:** 2026-08-18

**Authors:** Luis Victor-Gallardo, Andrey Salazar-Vargas, Laura González-Sanabria, Jeeno Soa George, Jairo Quirós-Tortós

**Affiliations:** a Climate Lead Group (CLG), San José, Costa Rica; b School of Social Sciences and Humanities, Loughborough University, Loughborough, United Kingdom

**Keywords:** Decision support, Deep uncertainty, Exploratory modelling and analysis, Energy systems planning, Uncertainty quantification, Computational experiment management

## Abstract

Long-term energy planning under deep uncertainty benefits from stress-testing strategies across many plausible futures. OSeMOSYS-RDM is an open-source, reproducible workflow that couples Robust Decision Making (RDM) with the Open Source energy Modeling System (OSeMOSYS). It automates uncertainty sampling (Latin Hypercube Sampling, LHS), ensemble model execution across multiple solvers, standardized post-processing, and scenario discovery using the Patient Rule Induction Method (PRIM). The workflow is configuration-driven (Excel and YAML), supports workflow reproducibility via Data Version Control (DVC), and produces shareable inputs/outputs for transparent, repeatable studies. Although developed for energy-system models, it can be applied to any OSeMOSYS-encoded system (e.g., land use or industrial processes). This MethodsX article provides a standalone description of the architecture, configuration interface, and outputs to enable reuse and adaptation by the OSeMOSYS community. The workflow automates the quantitative core of an RDM study (experimental design, ensemble execution, and scenario discovery) and is intended to support -not replace- participatory engagement with decision makers through which a full RDM analysis is framed and deliberated.

• OSeMOSYS-RDM uses Excel and YAML configuration files to define uncertainties, experiments, and outputs without modifying the core workflow code.
• It automates Latin Hypercube Sampling, batch and parallel solver execution (GLPK, CBC, CPLEX, and Gurobi), standardized post-processing into shareable datasets, and DVC-based pipeline execution for reproducible runs.
• The method is demonstrated on a multisectoral Uganda model and a Costa Rica transport decarbonization application.

## Graphical abstract

**Figure:**
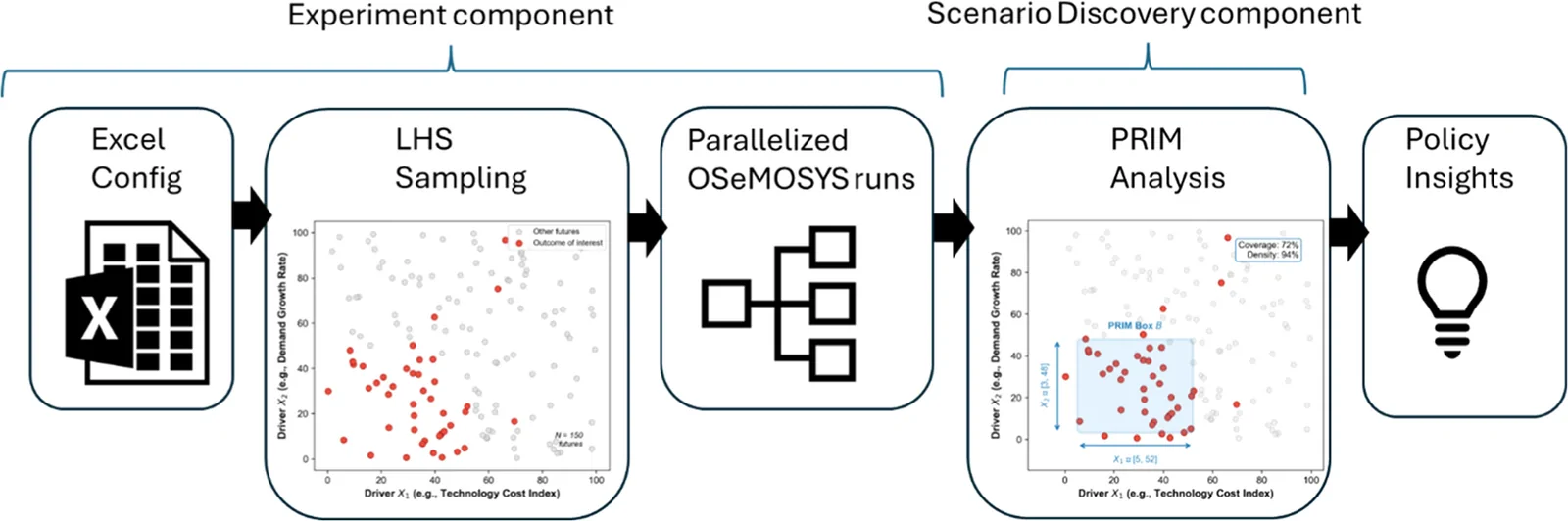

## Specifications table

**Table:** 

**Subject area**	Energy
**More specific subject area**	*Long-term energy-systems modelling and planning under deep uncertainty*
**Name of your method**	*OSeMOSYS-RDM*
**Name and reference of original method**	*Robust Decision Making (RDM)* [1]*; OSeMOSYS* [2]*; Patient Rule Induction Method (PRIM)* [3]
**Resource availability**	*GitHub (software):*https://github.com/clg-admin/osemosys-rdm

## Background

Long-term energy system planning under deep uncertainty requires strategies that remain acceptable across many plausible futures, rather than relying on a single “most likely” forecast. Decision Making under Deep Uncertainty (DMDU) frameworks such as Robust Decision Making (RDM) support this by stress-testing candidate strategies across large ensembles, identifying vulnerabilities, and iterating toward robust choices [1,4]. In its complete form, RDM is an iterative and participatory process in which analysts and decision makers jointly frame the analysis, agree on strategies, uncertainties, and performance metrics, and deliberate over the results; the workflow presented here operationalizes the quantitative experimentation within that cycle. RDM builds on Exploratory Modeling, which uses computational experiments to explore uncertainty spaces and discover system behaviors relevant to decisions [5]. Scenario discovery methods, including the Patient Rule Induction Method (PRIM), identify combinations of uncertain factors associated with policy-relevant outcomes [3,6].

In energy system optimization models (ESOMs), uncertainty assessment is often limited to a small number of deterministic sensitivity cases or, when probabilistic/stochastic approaches are used, requires specifying probability distributions that may be difficult to justify under deep uncertainty [7,8]. This is the case despite a substantial methodological literature on uncertainty analysis, exploratory modelling, and global sensitivity analysis for ESOMs [[9], [10], [11]]. As a result, a practical gap remains between available DMDU methods and the day-to-day workflows of many open-source ESOM practitioners.

OSeMOSYS is a widely used open-source ESOM designed for transparent, accessible modelling, with a growing global user and contributor community [2,12,13]. Recent systematic reviews of the OSeMOSYS literature identify uncertainty evaluation as an emerging and under-developed research direction, pointing to the need for reusable workflows that lower the barrier to robust analysis [14]. While the OSeMOSYS ecosystem includes data and interface tooling that improves model construction and reproducibility (e.g., MUIO, otoole, clicSAND) [[15], [16], [17]], and general-purpose exploratory modelling toolkits exist (e.g., EMA Workbench, Rhodium, OpenMORDM) [5,[18], [19], [20]], connecting these toolkits to OSeMOSYS inputs and outputs remains a non-trivial integration task. As a result, implementing RDM with OSeMOSYS has historically required substantial manual effort: generating parameter samples, modifying data files for each future, executing optimization runs, parsing and aggregating outputs, and applying scenario discovery techniques. This workflow is error-prone and difficult to reproduce, share, or port across OSeMOSYS-based domains.

This paper presents OSeMOSYS-RDM, a generalized and reusable workflow that operationalizes RDM for OSeMOSYS-based models [2]. The design is informed by ESOM best-practice guidance emphasizing transparency, reproducibility, and uncertainty communication [10,11], and aligns with open energy modelling principles [21]. OSeMOSYS-RDM includes the following elements:

(1) Configuration-driven design: All model-specific parameters are declared in external configuration files (Excel/YAML), reducing the need to modify source code for new applications.
(2) It is available for GLPK, CBC, CPLEX, and Gurobi solvers.
(3) It integrates the Data Version Control (DVC) python library that facilitated reproducibility [22].
(4) It integrates a scenario discovery library to identify parameter ranges associated with outcomes of interest [3,6].
(5) It parallelizes scenario generation and configurable batch sizes for different computational environments.
(6) OSeMOSYS-RDM is designed to be portable across OSeMOSYS applications, including energy-only and multi-domain models (e.g., land-use, industry, waste) that follow the OSeMOSYS architecture [2,13].

## Method details

**Fig. 1:**
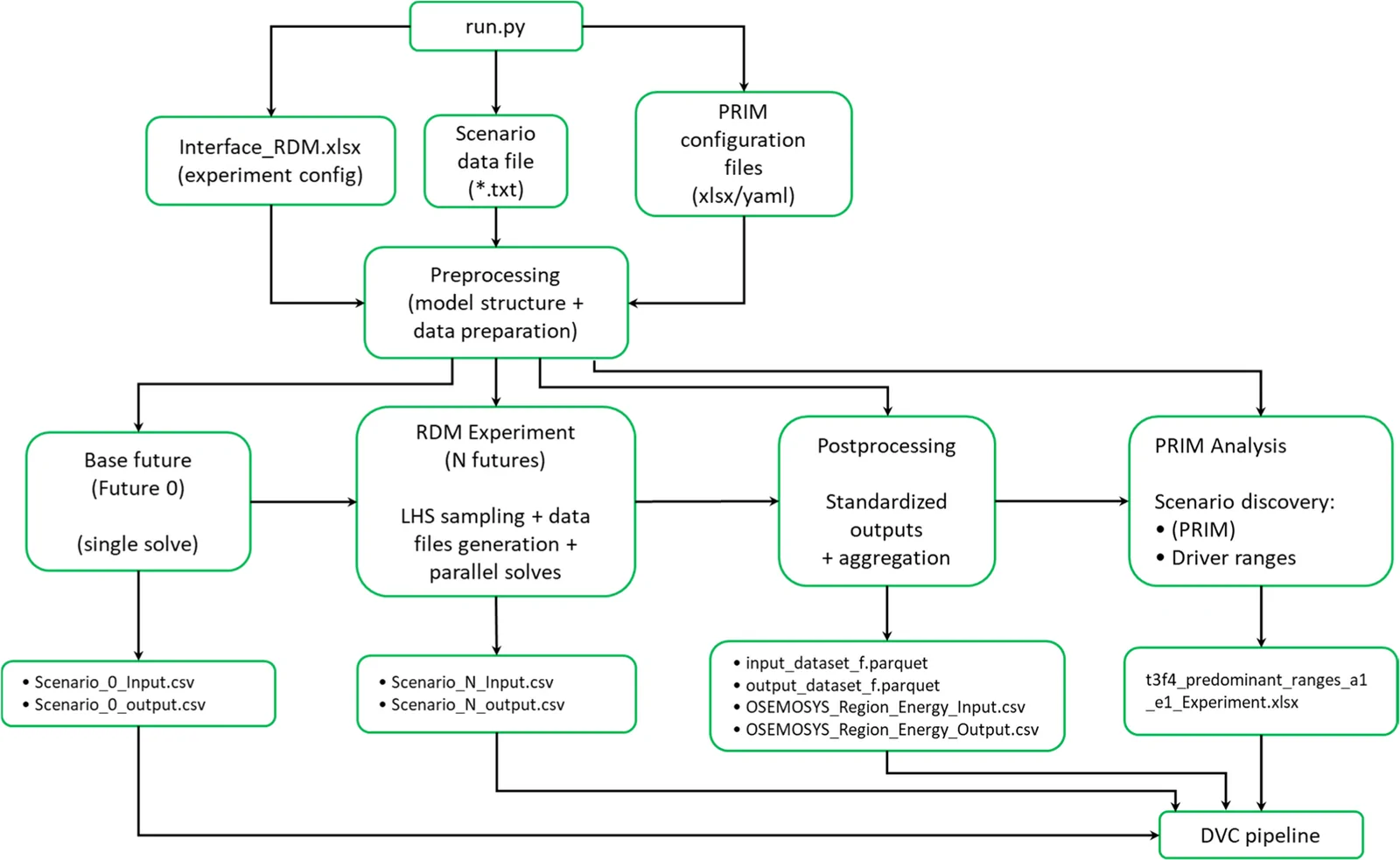
OSeMOSYS-RDM pipeline architecture. User inputs (Interface_RDM.xlsx, scenario data files, and PRIM configuration files) feed a five-stage workflow: preprocessing, Base Future (Future 0) execution, RDM experiment generation and parallel solves across N futures (via LHS sampling and data-file generation), postprocessing with standardized outputs and aggregated datasets, and PRIM scenario discovery to identify driver ranges. Reproducibility is supported through a fixed random seed and DVC pipeline caching (skipping unchanged stages), with key intermediate artifacts shown beneath each stage.

Having established the need for a reproducible, configuration-driven workflow that integrates ensemble generation with scenario discovery, we now describe how OSeMOSYS-RDM operationalizes this approach in practice. The workflow involves four steps (see Fig. 1):

(1) Configure the experiment parameters and uncertainty specifications in an Excel interface (Interface_RDM.xlsx);
(2) Navigate to the repository directory from the command line;
(3) Execute the pipeline using python run.py exp for ensemble generation, python run.py prim for scenario discovery, or python run.py rdm for both sequentially; and
(4) Retrieve outputs from the src/Results/ directory, which contains consolidated CSV and Parquet files.

The repository ships with a self-contained example model and configuration that constitute a minimum working example. The conda environment installs the open-source GLPK and CBC solvers automatically; GLPK is required in all cases because it converts the MathProg formulation into the LP file passed to the selected solver, which by default is the open-source CBC.

Upon execution, the script automatically verifies the Conda environment, installs missing dependencies, initializes Data Version Control (DVC), and runs each pipeline stage with automatic caching — unchanged stages are skipped on subsequent runs, reducing computation time during iterative experimentation. A beginner-friendly guide with annotated screenshots is available in the project documentation (refer to Supplementary Materials section). The following subsections detail the design philosophy, system architecture, configuration options, and analytical components that underpin this workflow.

### Design philosophy

The design prioritizes three principles:

• **Accessibility**: Users configure experiments through Excel spreadsheets rather than code modification
• **Reproducibility**: Pipeline execution is managed by DVC, ensuring consistent results across environments [22]. The automated pipeline is useful mainly to reproduce a project. However, if the user wants to create a full project, they will most likely need to execute the workflow script by script.
• **Flexibility**: Modular architecture supports different solvers, uncertainty specifications, and analysis configurations

OSeMOSYS-RDM targets the GNU MathProg implementation of OSeMOSYS and does not modify the mathematical formulation. The reference implementation is tested against the equation set distributed with MUIO v5.3. Users working with other OSeMOSYS implementations (e.g., Pyomo, PuLP, GAMS) can adopt the workflow conceptually but may need adapters for model execution and output parsing [15].

### System structure

**Fig. 2:**
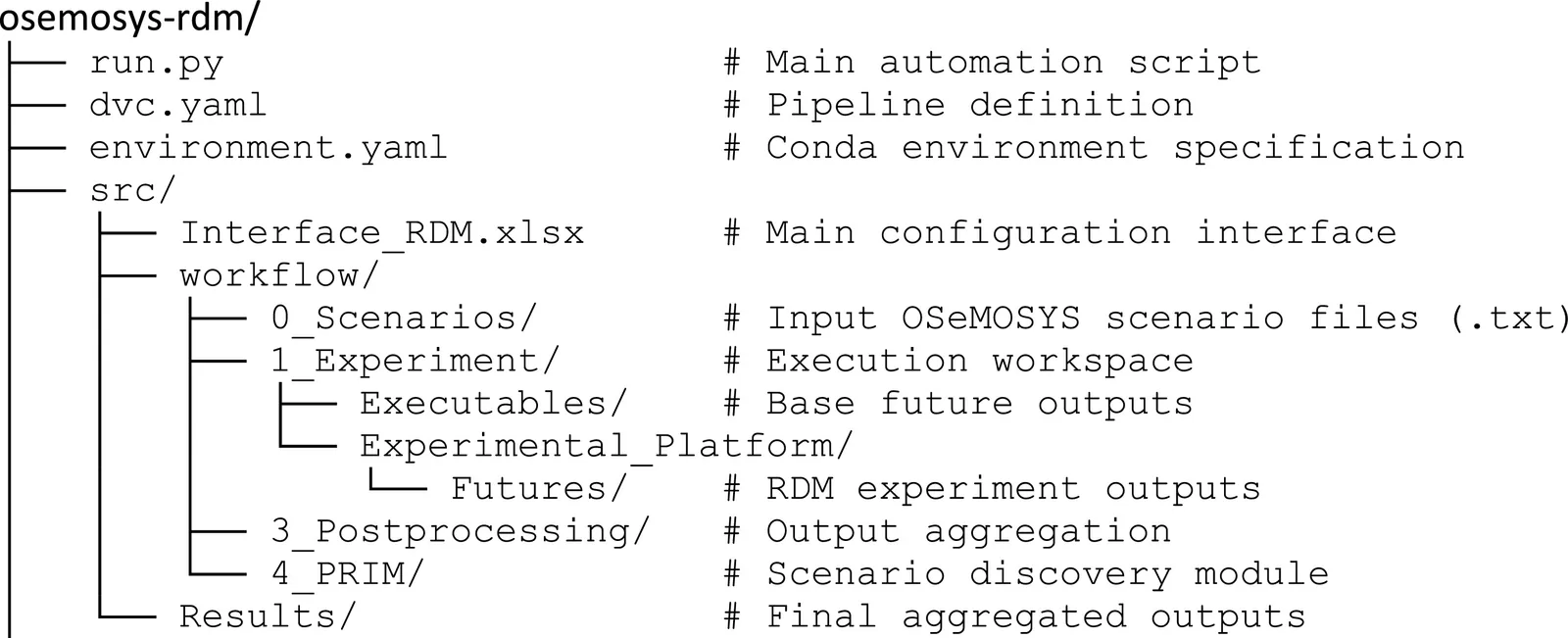
OSeMOSYS-RDM directory tree.

OSeMOSYS-RDM organizes files in a standardized layout that separates source code, configuration files, and outputs (see directory tree below) (Fig. 2).

### Configuration interface

The Excel interface contains four primary sheets:

**Setup Sheet** - Global experiment parameters:

**Table:** 

**Parameter**	**Description**	**Example**
Timeslices_model	Number of time slices in OSeMOSYS model	48
Run_Base_Future	Execute baseline scenario	Yes/No
Run_RDM	Execute RDM experiment	Yes/No
Number_of_Runs	Number of futures to generate (N)	500
Solver	Optimization solver	glpk/cbc/cplex/gurobi
Parallel_Use	Simultaneous parallel runs	8
Region	Model region identifier	Kenya

**Uncertainty_Table Sheet** - Defines uncertain parameters:

**Table:** 

**Column**	**Description**
X_Num	Unique uncertainty identifier
X_Category	Grouping label
X_Mathematical_Type	Interpolation method (see Section 2.4.2)
Explored_Parameter_of_X	Target type (Final_Value, Multiplier, etc.)
Exact_Parameters_Involved_in_Osemosys	OSeMOSYS parameter name(s)
Involved_First/Second/Third_Sets_in_Osemosys	Set elements to modify
Min_Value / Max_Value	Uncertainty range bounds
Initial_Year_of_Uncertainty	Year when uncertainty begins
Involved_Scenarios	Applicable scenarios

**Params_Sets_Vari Sheet** - Maps parameters to their index sets, ensuring correct data structure handling.

**To_Print Sheet** - Specifies which output parameters to extract from solver results.

The PRIM module uses additional configuration files in src/workflow/4_PRIM/:

prim_structure.xlsx: Defines outcomes and drivers for scenario discovery
prim_files_creator_cntrl.xlsx: Links experiments to analyses and defines time periods
PRIM_t3f2.yaml: Runtime parameters including scenario names and file patterns
Population.xlsx: Population data for per-capita normalization

Additionally, the OSeMOSYS components has two configurable modes from an Excel controller called Interface_RDM.xlsx:

**Run_Base_Future**: Executes a single baseline scenario (Future 0) for OSeMOSY model validation-

**Run_RDM**: Generates and analyzes N futures using Latin Hypercube Sampling.

These two can be activated at the same time, and they will run sequentially.

### Scenario generation

#### Latin hypercube sampling

OSeMOSYS-RDM generates futures using Latin Hypercube Sampling (LHS), implemented using pyDOE [23]. LHS is a stratified sampling technique that ensures efficient coverage of the uncertainty space: for N desired samples, each parameter's uncertainty range is divided into N equal intervals, and exactly one sample is drawn from each interval. This guarantees that every region of every parameter's distribution is represented, providing better space-filling coverage than simple random sampling with the same number of runs. The sampled values are then mapped to the corresponding parameter ranges and applied to the base scenario data. A fixed random seed ensures reproducibility across runs.

For OSeMOSYS-RDM, the sampling process:

1. Generate LHS matrix: hypercube = lhs(P, samples=N)
2. Transform samples to parameter ranges using inverse CDF: value = scipy.stats.uniform.ppf(sample, min_value, max_value - min_value)
3. Apply sampled values to base scenario data

A fixed random seed (for example, np.random.seed(555)) ensures reproducibility across runs.

#### Parameter perturbation methods

OSeMOSYS-RDM supports five interpolation methods for applying uncertainty to time-series parameters:

**Time_Series (Non-linear)**: Adjusts the final-year value by the sampled multiplier, with smooth non-linear interpolation from the uncertainty start year:

new_value[y] = base_value[y] × f(y, multiplier, initial_year, final_year)

**Constant**: Maintains the value at the uncertainty start year throughout the remaining horizon.

**Linear**: Applies linear interpolation between the uncertainty start year and the adjusted final value.

**Logistic**: Applies S-curve (logistic) interpolation for technology adoption or phase-out patterns.

**Time slices Curve**: Modifies sub-annual demand profiles by selecting from predefined load shape curves.

For further details, refer to Supplementary material S4, which contains complete follow-through examples of the parameter perturbation methods implemented in OSeMOSYS-RDM.

#### Data file generation

For each future f and scenario s, OSeMOSYS-RDM:

• Deep-copies the base scenario data structure
• Applies sampled perturbations to specified parameters
• Generates GNU MathProg format data files (.txt)
• Saves input parameter snapshots (.parquet and .csv)

Memory management is critical for large experiments. OSeMOSYS-RDM chunks the scenario dictionary into pickle files, loading only the required futures for each parallel batch.

### Model execution

An example that executes the reproducible DVC pipeline is available in the project’s documentation at https://osemosys-rdm.readthedocs.io/en/latest/results/index.html#. The subsections below explain the features of the workflow: solvers, parallelism, and output processing.

#### Solver abstraction

OSeMOSYS-RDM abstracts solver differences through a unified execution layer:

**Table:** 

**Solver**	**Data Preparation**	**Execution Command**
GLPK	Direct MathProg	glpsol -m model.txt -d data.txt -o output.txt
CBC	Convert to LP	glpsol…–wlp output.lp then cbc output.lp solve -solu output.sol
CPLEX	Convert to LP	glpsol…–wlp output.lp then cplex -c "read output.lp" "optimize" "write output.sol"
Gurobi	Convert to LP	glpsol…–wlp output.lp then gurobi_cl ResultFile=output.sol output.lp

#### Parallel execution

Model runs are parallelized using Python's multiprocessing module. The Parallel_Use parameter controls batch size:

for batch in range(num_batches):

processes = []

for run in range(batch_start, batch_end):

p = mp.Process(target=execute_model, args=(run, …...))

processes.append(p)

p.start()

for p in processes:

p.join()

This approach balances memory usage against execution speed, as each process loads its required data independently.

#### Output processing

Solver outputs are parsed using format-specific handlers that extract decision variable values. Results are standardized to a common schema:

**Table:** 

**Column**	**Description**
Future.ID	Future identifier (0 to N)
Strategy.ID	Scenario identifier
Technology / Commodity / Emission	OSeMOSYS set elements
Year	Model year
[Parameter]	Variable values

Outputs are stored in Parquet format for efficient storage and fast subsequent analysis.

### Postprocessing

The postprocessing stage aggregates results across all futures into consolidated files:

OSEMOSYS_{Region}_Energy_Input.csv: Aggregated input parameters
OSEMOSYS_{Region}_Energy_Output.csv: Aggregated output variables

Configuration via config_concatenate.yaml specifies which parameters to include and any transformations to apply.

### Scenario discovery (PRIM)

#### PRIM methodology

The Patient Rule Induction Method (PRIM) is a scenario discovery algorithm that identifies hyper-rectangles (“boxes”) in the uncertainty space that are highly associated with cases meeting a policy-relevant outcome criterion (e.g., costs exceeding a pre-defined threshold) [3,6]. PRIM iteratively, the scenario ensembles to balance box density (the proportion of cases within the box that meet the criterion, i.e., box 'purity') and coverage (the proportion of all cases meeting the criterion that fall within the box, i.e., how much of the outcome the box 'explains'). OSeMOSYS-RDM implements PRIM analysis through four sequential scripts:

**Table:** 

**Order**	**Script**	**Function**
1	t3f1_prim_structure.py	Parses configuration, creates analysis structure
2	t3f2_prim_files_creator.py	Extracts metrics from RDM outputs
3	t3f3_prim_manager.py	Executes PRIM algorithm used in the prim python library [24].
4	t3f4_range_finder_mapping.py	Maps results to interpretable ranges

In OSeMOSYS-RDM, PRIM is applied after the ensemble has been generated and post-processed into standardized input–output datasets. PRIM takes as input the full set of sampled uncertainties from the RDM experiment and identifies a subset (along with specific ranges of their values) that is statistically associated with a specified outcome (e.g., system costs exceeding a threshold). This subset constitutes the 'drivers' of that outcome, named as drivers because PRIM has revealed them to be explanatory. The method then allows us to express these relationships as interpretable rules specifying the ranges of driver values that predict the outcome of interest. PRIM expresses these relationships as rule-like driver ranges that summarize “risk” or “success” cases as defined by the selected thresholds.

**Fig. 3:**
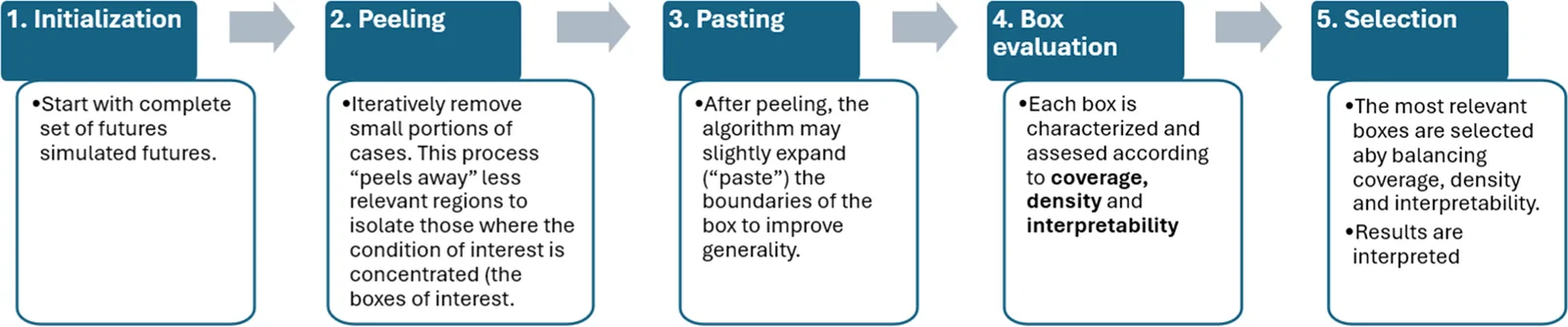
PRIM scenario discovery workflow used in OSeMOSYS-RDM. PRIM identifies interpretable regions (“boxes”) in the uncertainty space that are associated with outcomes of interest by iteratively restricting ranges of uncertain drivers (peeling/pasting) and evaluating candidate boxes using coverage (share of target cases captured), density (concentration of target cases inside the box), and interpretability (parsimony of driver ranges). Outcomes can be defined using distribution-based thresholds (e.g., upper/lower quartiles) to classify “risk” and “desirable” cases.

Before applying PRIM, the user must define the outcome classes that distinguish 'cases of interest' from other futures. This can be done using absolute thresholds (e.g., classifying futures where total system cost exceeds a specific value) or quantile-based thresholds (e.g., classifying futures in the upper quartile of costs). The former is useful when policy-relevant cutoffs are known in advance; the latter is useful for exploratory analysis when the goal is to characterize the tails of the outcome distribution. Fig. 3 summarizes the PRIM workflow as implemented in this repository, including (i) the iterative box search, and (ii) the reporting of predominant driver ranges for each outcome and time period.

Once boxes are selected, the analyst can examine the predominant driver ranges that define each box - that is, the specific intervals of uncertainty values associated with the outcome of interest. In OSeMOSYS-RDM, this analysis is performed separately for each outcome metric and time period.

#### Outcome and driver specification

Users configure two elements in prim_structure.xlsx. First, they specify the outcomes to be explained; metrics such as total system cost, technology-specific emissions, or renewable energy share; along with the thresholds that define cases of interest (e.g., 'risk' as upper quartile, 'desirable' as lower quartile). Second, they specify the candidate input variables -the uncertain parameters from the RDM experiment- that PRIM should evaluate as potential explanatory factors. PRIM then analyzes these candidates and identifies which subset qualifies as drivers: those whose value ranges are statistically associated with the specified outcomes.

**Outcomes** - Metrics to analyze, such as:

Total system cost
Technology-specific emissions
Renewable energy share
Per-capita metrics (using population normalization)

**Candidate input parameters -** Uncertain parameters from the RDM experiment that PRIM will evaluate as potential drivers. These are user-defined and case-specific; examples might include:

• SpecifiedAnnualDemand for sectoral/fuel demands (e.g., residential, transport fuels)
• CapitalCost for technology investment costs (e.g., electric transport, ICE transport, industry)
• FixedCost for annual fixed O&M costs (e.g., crops/land-use, transport technologies)
• VariableCost for variable O&M and commodity/import costs (e.g., imported fuels, agricultural commodities)
• OperationalLife for technology lifetimes
• InputActivityRatio / OutputActivityRatio (and related efficiency parameters) for technology performance
• Capacity constraints such as TotalCapacityAnnualUpperLimit / TotalCapacityAnnualLowerLimit (and related build/availability limits)
• Emissions factors such as EmissionActivityRatio

For each metric-outcome combination, PRIM generates multiple candidate boxes with varying coverage, density, and interpretability. The user can select one box per metric-outcome to serve as the final result, applying criteria suited to the analysis goals. For example, selecting the box with the greatest number of drivers while maintaining at least 70% coverage. To illustrate: for a metric such as 'system cost,' PRIM produces one set of candidate boxes for 'desirable' outcomes (e.g., costs below a threshold) and a separate set for 'risk' outcomes (e.g., costs above a threshold). The analyst then selects one box from each set, yielding two final results for that metric - one characterizing condition associated with desirable costs, the other with risk costs.

#### Metric formulations

OSeMOSYS-RDM supports complex metric calculations:

**Table:** 

**Formula Type**	**Description**	**Example**
sum	Sum across sets and years	Total emissions
average	Mean across sets	Average capacity factor
share	Ratio of subset to total	Renewable generation share
cumulative	Period sum	Cumulative investment
direct	Single value extraction	Final year capacity
wrt_bau	Difference from baseline	Cost increase vs. BAU

#### Output interpretation

PRIM results are exported to t3f4_predominant_ranges_*.xlsx with columns:

**Table:** 

**Column**	**Description**
Driver	Uncertain parameter name
Min_Norm / Max_Norm	Normalized range (0–1) defining the "box"
Outcome_Type	"Desirable" or "Risk"
Metric	Outcome analyzed
Period	Time period of analysis

These ranges indicate which parameter combinations lead to favorable or unfavorable outcomes. Within a single selected box (one metric-outcome combination), the identified driver ranges are concurrent: all conditions must be met simultaneously to predict the outcome. Across different metric-outcome combinations, however, results are independent - a given driver may have different, even opposing, ranges associated with different outcomes. Users should therefore interpret each metric-outcome result on its own terms before comparing across results to identify synergies or trade-offs. More detail about the interpretation of concurrency is in Supplementary Material S5. Box quality metrics are also produced by the workflow: for every candidate box evaluated during scenario discovery, coverage (the share of all cases meeting the outcome criterion that fall within the box) and density (the share of cases within the box that meet the criterion) are computed and used internally to select the best box. The selected box's coverage, density, and their average are reported in the scenario-discovery output file sd_ana_<analysis>_*exp_*<experiment>.csv alongside its driver ranges.

## Method validation

Method validation is provided through an end-to-end demonstration on a multisectoral national model for Uganda encoded using the OSeMOSYS architecture. The validation objective is to confirm that the workflow executes reproducibly on a model that includes non-energy components expressed within the standard OSeMOSYS data structure, including transport and industry (with mining-related processes) alongside agriculture and land-use representations.

Development provenance is documented to clarify the relationship between prior case-study work and the current implementation. The workflow described here was prototyped in earlier Costa Rica decarbonization work using a legacy scripting approach [25]. The present repository is a refactored, configuration-driven and reproducible implementation; therefore, the Uganda demonstration (Section `Multisectoral Uganda demonstration') is used as the primary validation for this MethodsX article.

Validation data are provided in the form of workflow inputs, execution records, and standardized outputs generated from a baseline run (Future 0) and an exploratory ensemble of 20 futures created via Latin Hypercube Sampling. Uncertain inputs are represented as normalized scalars and grouped into (i) demand drivers (e.g., fuel demand and residential demand) and (ii) cost drivers spanning transport, industry, imported fuels, agricultural commodities, and land-use and transport technologies (capital, fixed, and variable cost parameters). The workflow compiles run metadata and outputs into a standardized experiment database suitable for subsequent analysis.

To validate the scenario discovery component, PRIM is configured to analyze cost and emissions outcomes by defining “desirable” and “risk” cases using percentile-based thresholds (e.g., lower and upper quartiles) and evaluating driver ranges over multiple time periods (2021–2030, 2031–2040, 2041–2050). Supplementary materials S6 and S7 provide examples of application, including results, for the experimentation and scenario discovery components, respectively.

To ensure that the pipeline can be verified quickly by any user, the default branch of the repository bundles an energy-system model for South Africa as the experiment-stage example, which is smaller and faster to solve than the Uganda model reported in Section 3.1. The experiment results underlying the Uganda scenario-discovery demonstration are committed to the repository, so the PRIM stage reproduces the reported results directly with python run.py prim without re-solving the ensemble. The complete Uganda experiment configuration is maintained on a dedicated uganda branch, where the full ensemble can be re-solved at a considerably longer runtime. In verification runs of the default branch, ensemble generation (python run.py exp) completed in approximately four minutes and scenario discovery (python run.py prim) in approximately one minute, with both stages executed sequentially (python run.py rdm) completing in approximately five minutes.

### Multisectoral Uganda demonstration

To check that OSeMOSYS-RDM functions reliably on a model that extends beyond a conventional “energy-only” ESOM, we applied the workflow to a multisectoral national model for Uganda encoded using the OSeMOSYS architecture. In addition to the energy system, the model represents transport, industry (including mining-related processes), and agriculture/land-use components. As is standard for OSeMOSYS applications, the model operates on an annual time step over the 2021–2050 analysis horizon, with each year subdivided into time slices (eight in this application) to represent sub-annual variation in demand and supply.

Using the Excel configuration interface (Interface_RDM.xlsx), we specified a baseline run (Future 0) and an exploratory ensemble of 20 futures generated via Latin Hypercube Sampling. Uncertainties were represented as normalized scalars and organized into two groups: (i) demand drivers (e.g., fuel demand and residential demand) and (ii) technology and commodity cost drivers (capital, fixed, and variable costs) spanning transport, industry, imported fuels, and agricultural commodities.

The workflow executed the ensemble runs and compiled outputs into a standardized experiment database. Post-processing then computed selected performance metrics (system costs and emissions) for each run. Scenario discovery was performed using the PRIM module to identify combinations of uncertain inputs associated with “desirable” and “risk” outcomes, defined using percentile-based thresholds, across three time periods (2021–2030, 2031–2040, 2041–2050) for the selected metrics.

The resulting PRIM outputs (driver-range tables and predominant-range summaries) illustrate that the workflow can surface cross-sector drivers of performance and vulnerability. In this demonstration, transport technology costs and fuel-demand growth interact with non-energy-sector parameters (e.g., agricultural cost assumptions) in ways that influence the likelihood of high-emissions and/or high-cost futures. As the purpose of this MethodsX article is to document the reusable workflow, the examples using the Uganda model are in Supplementary Materials S6 and S7.

A key feature of the Uganda demonstration is that it was configured without modifying the workflow source code. Adapting OSeMOSYS-RDM to this application required only (a) defining uncertainties and experiment settings in Interface_RDM.xlsx and (b) providing an OSeMOSYS model instance consistent with the expected input/output conventions. This serves as a practical efficiency check relative to application-specific, one-off scripting approaches.

Earlier OSeMOSYS ensemble applications have often been disseminated as collections of run-specific input files and outputs. For example, Moksnes et al. archive 324 OSeMOSYS data files (one per run), an accompanying Access database containing results from the 324 runs, and link to a separate repository for PRIM analysis [26]. OSeMOSYS-RDM follows the same principle of transparency, but emphasizes sharing the experiment specification (uncertainty table, sampling seed, and configuration files) together with an automated pipeline so that ensembles and analyses can be regenerated without manually assembling hundreds of model instances.

### Costa Rica application

The workflow was demonstrated using a national decarbonization case study for Costa Rica, focusing on transport–power sector interactions under deep uncertainty [25]. This application is included to illustrate how an end-to-end RDM experiment is structured in practice-uncertainty sampling, batch model solving, post-processing, and PRIM-based scenario discovery-and to show how typical OSeMOSYS study assumptions map onto the configuration interface. In this case, uncertain inputs included electricity and transport demand growth, technology cost assumptions (e.g., solar PV, wind, storage, and transport technologies), and fuel price trajectories and other exogenous drivers. Scenario discovery was then used to identify combinations of uncertain conditions associated with selected outcomes of interest (e.g., system cost, emissions, and reliability-related metrics). The Costa Rica case study reported in [25] was implemented using an earlier, study-specific version of the workflow; the present MethodsX article documents a generalized and updated implementation informed by that experience.

### Generalizability and recommended repository structure

To keep the workflow reusable and publication-ready, we recommend separating the core workflow code from case-specific model data and assumptions. The core repository (osemosys-rdm) should contain the generic pipeline, configuration templates, documentation, and a minimal example. Each application should be maintained in a separate case repository (e.g., osemosys-rdm-cr, osemosys-rdm-ug) that stores model inputs, scenario assumptions, and analysis configurations, and pins these to a specific osemosys-rdm release (or commit) without modifying the core workflow code. In practice, this means maintaining (i) a core repository for code and templates, (ii) one repository per case study for inputs and configuration, and (iii) optional DVC remotes to version and retrieve the input/output artifacts required for end-to-end reproducibility [22]. At present, the repository uses dedicated branches to separate case studies (e.g., the South Africa example on the default branch and the full Uganda experiment on the uganda branch). A planned refinement is to consolidate case-specific configurations into clearly named folders within a single branch (e.g., cases/South-Africa/, cases/Uganda/), so that users can switch between applications by selecting a configuration directory rather than checking out a different branch.

OSeMOSYS-RDM is distributed as open-source software under the Apache 2.0 license and is available in the project repository, together with documentation and example configurations. Case-study replication can be supported by maintaining separate, version-pinned case repositories that store model inputs and analysis configuration.

## Limitations

OSeMOSYS-RDM is designed for repeated execution of OSeMOSYS models formulated as linear programs (LPs). Applications that require mixed-integer formulations are not supported in the current implementation without additional development. The workflow enables large ensemble experiments, but computational requirements can become substantial for high number of samples, long time horizons, or large models, and performance can vary across solvers. Uncertainty sampling reflects user-specified parameter ranges with a uniform distribution. The workflow does not assess whether these assumptions are appropriate for a given decision context. Scenario discovery using PRIM depends on the definition of outcomes of interest, thresholds, and analysis settings, and results are sensitive to these choices and to ensemble size. Finally, while DVC supports experiment tracking and caching, full reproducibility also depends on preserving the exact versions of the workflow, the specific OSeMOSYS model instance, and the input datasets used. In addition, OSeMOSYS-RDM covers the quantitative procedure of RDM: a complete RDM study is an iterative and participatory process in which decision makers help frame the problem, select strategies, uncertainties, and performance metrics, and deliberate over vulnerabilities and trade-offs. These qualitative activities are not automated by the workflow and remain the responsibility of the analysis team.

## Ethics statements

This study did not involve human participants, animal experiments, or the collection/analysis of personal data. All results are produced from computational experiments using openly available models and/or secondary datasets. Therefore, ethical approval and informed consent were not required.

## Declaration of generative AI and AI-assisted technologies in the manuscript preparation process

During the preparation of this work the author(s) used Claude (Anthropic) and ChatGPT (OpenAI) in order to assist with technical writing and restructuring of method descriptions (including the OSeMOSYS-RDM workflow and PRIM scenario discovery), code and documentation cross-checking for consistency, drafting/refining repository documentation, and formatting references where needed. After using these tools/services, the authors reviewed and edited the content as needed and take full responsibility for the content of the published article.

## Supplementary material *and/or* additional information [OPTIONAL]

The github repository of OSeMOSYS-RDM is https://github.com/clg-admin/osemosys-rdm and the documentation is in https://osemosys-rdm.readthedocs.io/en/latest/results/index.html. The following supplementary materials are available:

S1: Complete Interface_RDM.xlsx template with example configuration, including an uncertainty table.

S2: User Guide for OSeMOSYS-RDM

S3: User Guide for Using the PRIM module

S4: Working examples for the parameter perturbation methods

S5: Concurrency analysis

S6: OSeMOSYS-RDM Example: Experiment Component (demonstration output)

S7: Uganda PRIM Summary Report (demonstration outputs)

## CRediT authorship contribution statement

**Luis Victor-Gallardo:** Conceptualization, Methodology, Software, Writing – original draft. **Andrey Salazar-Vargas:** Conceptualization, Software, Validation. **Laura González-Sanabria:** Writing – review & editing. **Jeeno Soa George:** Writing – review & editing. **Jairo Quirós-Tortós:** Funding acquisition, Supervision, Writing – review & editing.

## Declaration of competing interest

The authors declare that they have no known competing financial interests or personal relationships that could have appeared to influence the work reported in this paper.

## Data Availability

Code and an illustrative sample model are available in the OSeMOSYS-RDM repository. The sample model is for demonstration only and is not an official model.

## Appendix Supplementary materials

Image, application 1
