# Supplementary material for: OSeMOSYS-RDM: A reproducible workflow for robust decision making with OSeMOSYS models
Source: MethodsX. 2026 Aug 18;17:104102. doi: 10.1016/j.mex.2026.104102 (PMC13543070; doi:10.1016/j.mex.2026.104102)
Supplement: Supplementary file 1 [file mmc1.zip › S2_Guide OSeMOSYS_RDM.html]

RDM Model - User Guide


# AFR\_RDM Model - User Guide


### 📚 Contents

#### Introduction

Overview
Prerequisites

#### Initial Setup

1. Folder Structure
2. Workflow Folder
3. Scenarios Folder
4. Return to Main

#### Interface RDM

5. Open Interface\_RDM
6. Uncertainty\_Table Sheet
Column Configuration

#### Setup Configuration

7. Setup Sheet
Configuration Parameters
8. Save and Close

#### Execution

9. Open Spyder
10. Open Project
11. Load RUN\_RDM
12. Configure Execution
13. Run Process
Result Verification

#### PRIM Module

PRIM Configuration


## 📋 Overview

The AFR\_RDM (Africa Robust Decision Making) model is an essential tool
that allows for the evaluation of multiple scenarios and uncertainties in energy optimization models.
This guide provides step by step instructions for setting up and running RDM analyses.

## ✅ Prerequisites

| Component | Required Version | Status |
| --- | --- | --- |
| Python | 3.8 or higher | ✓ Required |
| Anaconda/Spyder | Latest version | ✓ Required |
| MOMF Framework | v2.0+ | ✓ Required |
| Microsoft Excel | 2016 or higher | ✓ Required |
| GLPK | v4.65 | ✓ Required |
| CBC | 2.7.5 (ideal) | ✓ Required |
| CPLEX | Any version | ⚬ Optional |
| Gurobi | Any version | ⚬ Optional |

## 1 Folder Structure

1

### Navigate to main folder

Go to the main project folder. In this case, the folder is named
src. This folder contains all the necessary structure
to run the RDM model.

## 2 Workflow Folder

2

### Access workflow

Double-click on the workflow folder.
This folder contains the model configuration files and scenarios.

## 3 Scenarios Folder

3

### Open 0\_Scenarios

Double-click on 0\_Scenarios.
This is where you will place all scenarios you want to analyze in the experiment.

⚠️ Important

Ensure all scenario files are properly formatted and contain the necessary parameters before continuing.

## 4 Return to Main Folder

4

### Go back to src

Navigate back to the main src folder to access the Interface\_RDM file.

## 5 Open Interface\_RDM

5

### Open Interface\_RDM file

Double-click on src\Interface\_RDM.xlsx
to open the experiment configuration. This Excel file contains all
necessary settings to run the RDM analysis.

## 6 Uncertainty\_Table Sheet

6

### Configure uncertainties

In the first sheet named **Uncertainty\_Table**, you will define all uncertainties
you want to evaluate in the experiment. Follow the column structure detailed in the next section.

## 📊 Column Configuration - Uncertainty\_Table

### Uncertainty table structure

The **Uncertainty\_Table** sheet must be configured with the following columns (A through N):

| Column | Field Name | Description | Example |
| --- | --- | --- | --- |
| A | **X\_Num** | Uncertainty number (ascending order) | 1, 2, 3... |
| B | **X\_Category** | Uncertainty category | Fuel Costs, Capital Cost |
| C | **X\_Plain\_English\_Description** | Brief description of the uncertainty | Changes in imported energy carriers |
| D | **Involved\_Scenarios** | Involved scenarios (separated by " ; ") | Scenario1 ; Scenario2 |
| E | **X\_Mathematical\_Type** | Mathematical type of the series:  • **Time\_Series**: Replicates original curve behavior  • **Constant**: Constant curve  • **Logistic**: Logistic curve  • **Linear**: Linear curve | Time\_Series |
| F | **Explored\_Parameter\_of\_X** | Parameter to explore | Final\_Value |
| G | **Initial\_Year\_of\_Uncertainty** | Starting year for uncertainty variations | 2025 |
| H | **Min\_Value** | Minimum variation value | 0.5 |
| I | **Max\_Value** | Maximum variation value | 1.5 |
| J | **Exact\_Parameters\_Involved\_in\_Osemosys** | Exact OSeMOSYS parameters (separated by " ; ") | VariableCost ; CapitalCost |
| K | **Involved\_First\_Sets\_in\_Osemosys** | Values of the first set used by the parameter (technologies or fuels) | MIMBIO ; MINCOA |
| L | **Involved\_Second\_Sets\_in\_Osemosys** | Values of the second set (if applicable per Params\_Sets\_Vari sheet) | BIO ; COA |
| M | **Involved\_Third\_Sets\_in\_Osemosys** | Values of the third set (if applicable per Params\_Sets\_Vari sheet) | TS01 ; TS02 |
| N | **Manipulation\_of\_Parameter** | Description of data manipulation according to X\_Mathematical\_Type | Multiply final value by explored parameter and interpolate |

💡 Important Notes about Sets

- To determine which sets apply to a parameter, check the **Params\_Sets\_Vari** sheet
- Each OSeMOSYS parameter column shows its required sets (excluding Region)
- Parameters highlighted in brown do not apply for this functionality
- If a parameter uses 2 sets: place first in column K, second in column L
- If a parameter uses 3 sets: place first in column K, second in column L, third in column M
- Always use exact format with spaces: `Value1 ; Value2 ; Value3`

## 7 Setup Sheet

7

### Navigate to Setup sheet

Go to the **Setup** sheet. In this sheet, you should only modify the cells highlighted in green.

## ⚙️ Configuration Parameters - Setup Sheet

### Editable green cells

In the Setup sheet, only modify the cells highlighted in green:

| Cell | Parameter | Description | Example Value |
| --- | --- | --- | --- |
| **A2** | Number\_of\_Runs | Number of futures to generate | 2 |
| **B2** | Scenario\_to\_Reproduce | **⚠️ DO NOT MODIFY - Fixed value** | **Experiment** |
| **C2** | Experiment\_ID | **⚠️ DO NOT MODIFY - Fixed value** | **1** |
| **D2** | Parallel\_Use | Number of parallel processes | 2 |
| **E2** | Timeslices\_model | Number of timeslices in the model | 48 |
| **F2** | Run\_Base\_Future | Execute base scenario | Yes/No |
| **G2** | Run\_RDM | Execute RDM process | Yes/No |
| **H2** | Solver | Solver for solving the RDM process | cbc/cplex/glpk/gurobi |
| **I2** | OSeMOSYS\_Model\_Name | OSeMOSYS model name | model.v.5.3.txt |
| **J2** | Region | Region name (use ISO-3 country code) | UGA/KEN |

⚠️ Critical Notes

- **DO NOT MODIFY** cells B2 (Scenario\_to\_Reproduce) and C2 (Experiment\_ID)
- If your PC shuts down during the process, reduce Parallel\_Use to 1
- For PCs with better specifications, you can experiment with higher parallel values
- Set Run\_Base\_Future to "Yes" when running for the first time or after model changes

## 8 Save and Close

8

### Save the Interface\_RDM file

Click **Save** to save all changes made to the Interface\_RDM.xlsx file,
then **Close** Excel.

## 9 Open Spyder

9

### Launch Spyder application

Open Spyder from your applications or Anaconda Navigator.
If any pop-up windows appear, click "No" to dismiss them.

## 10 Open Project Folder

10

### Navigate to src folder

In Spyder, click **File → Open** and navigate to your src folder.

## 11 Load RUN\_RDM Script

11

### Open src\RUN\_RDM.py

Select the file RUN\_RDM.py and click **Open** to load it in Spyder.

## 12 Configure Execution

12

### Set up Spyder configuration

Configure the execution environment:

1. Go to **Run → Configuration per file**
2. Select **"Run file with custom configuration"**
3. Select **"Execute in an external system terminal"**
4. Click **"Interact with the Python console after execution"**
5. Click **OK**

## 13 Run the RDM Process

13

### Execute the script

Click **Run** to start the RDM process. A terminal window will appear showing the execution progress.

Step 1 finished  
Step 2 finished  
Step 3 finished  
Step 4 finished  
Step 5 finished  
Step 6 finished  
Step 7 finished  
Step 8 finished  
GLPSOL: GLPK LP/MIP Solver, v4.65  
...  
Reading model section from ./workflow/osemosys\_code\_cloud.txt  
...  
OPTIMAL LP SOLUTION FOUND

## ✔️ Result Verification

### Process completed successfully

The process has finished correctly when you see the message:

Processing completed successfully  
#####################################  
The total time of the workflow has been: XXXX.XX seconds  
>>>

### Verify solutions

It's important to verify that the message **"OPTIMAL LP SOLUTION FOUND"**
appears for each future executed. The number of these messages should match the number
of futures configured in the setup.

✅ Final verification checklist

- Check that all futures have optimal solutions
- Verify output files in the Results folder
- Ensure there are no error messages in the console
- Confirm the total execution time is displayed

## 🔧 PRIM Module Configuration

### Objective

Establish the steps and artifacts necessary to reproduce PRIM execution (Excel inputs, Python scripts, and YAML files).

### Pipeline/Workflow Flow

**Execution Order to Replicate PRIM:**

#### 1. Input Data Preparation

- Complete src\workflow\4\_PRIM\t3b\_sdiscovery\Analysis\_1\prim\_structure.xlsx (Sequences and Outcomes sheets)
- Fill src\workflow\4\_PRIM\Population.xlsx file with demographic data
- Configure src\workflow\4\_PRIM\prim\_files\_creator\_cntrl.xlsx with control parameters
- Configure src\workflow\4\_PRIM\t3b\_sdiscovery\Units.xlsx with measurement units for drivers

#### 2. YAML Configuration

- Verify and adjust src\workflow\4\_PRIM\PRIM\_t3f2.yaml according to project needs

#### 3. Python Script Execution (in order)

- src\workflow\4\_PRIM\t3b\_sdiscovery\t3f1\_prim\_structure.py - Processes PRIM structure and generates prim\_files\_creator.pickle
- src\workflow\4\_PRIM\t3f2\_prim\_files\_creator.py - Creates PRIM files using experiment data
- src\workflow\4\_PRIM\t3b\_sdiscovery\t3f3\_prim\_manager.py - Executes PRIM analysis and generates results
- src\workflow\4\_PRIM\t3b\_sdiscovery\t3f4\_range\_finder\_mapping.py - Maps predominant driver ranges

#### 4. Results Interpretation

- Check the results in the file src\workflow\4\_PRIM\t3b\_sdiscovery\t3f4\_predominant\_ranges\_a1\_e1\_Experiment.xlsx.

📝 Important Note

To complete the "Input Data Preparation", "YAML Configuration" and "Results Interpretation" steps, follow the "Guide PRIM Module Configuration".

## 🎉 Process Complete

Congratulations! You have successfully configured and executed the RDM model.
The results are now available in the output folder for further analysis.
