# Supplementary material for: OSeMOSYS-RDM: A reproducible workflow for robust decision making with OSeMOSYS models
Source: MethodsX. 2026 Aug 18;17:104102. doi: 10.1016/j.mex.2026.104102 (PMC13543070; doi:10.1016/j.mex.2026.104102)
Supplement: Supplementary file 1 [file mmc1.zip › S3_Guide PRIM Module Configuration.html]

PRIM Module Configuration - Documentation


# PRIM Module Configuration

Complete Guide for Parameter Setup and Template Configuration


## Overview

**Objective:** Establish the steps and artifacts necessary to set parameters of the PRIM (Excel inputs and YAML files).

## Pipeline of Set Parameters

### Execution Order to Replicate PRIM:

1. **Input Data Preparation**
   - Complete `prim_structure.xlsx` (Sequences and Outcomes sheets)
   - Fill `Population.xlsx` file with demographic data
   - Configure `prim_files_creator_cntrl.xlsx` with control parameters
   - Configure `Units.xlsx` with measurement units for drivers
2. **YAML Configuration**
   - Verify and adjust `PRIM_t3f2.yaml` according to project needs

## Required Inputs

- `t3b_sdiscovery/Analysis_1/prim_structure.xlsx` (Sequences and Outcomes sheets)
- `Population.xlsx` (historical/projected population)
- `prim_files_creator_cntrl.xlsx` (execution control)
- YAML configuration file: `PRIM_t3f2.yaml`
- `t3b_sdiscovery/Units.xlsx` (unit definition per driver)

**Important Notes:**

- Maintain column names exactly as they appear in the templates
- Unused sheets in `prim_structure.xlsx` are: Indications, Qs\_and\_Notes, Command\_Desc, Set\_Matching\_4\_Param\_Mult

## Fill Templates

### 1. prim\_structure.xlsx

#### Sheet: Sequences

This sheet defines the complete PRIM analysis structure with drivers and outcomes.

**IMPORTANT:** When format requires semicolon separation, always use: space + semicolon + space (example: "value1 ; value2 ; value3")

#### Identification Columns

| Column Name | Explanation | Fill Format | Predefined Values | Example |
| --- | --- | --- | --- | --- |
| **Table\_ID** | Unique identifier for PRIM table | Positive integer | Sequential starting at 1 | 1 |
| **Level** | Hierarchical level of analysis | Integer | 1, 2, 3 | 1 |
| **Block\_ID** | Thematic block identifier | Same value as "Table\_ID" column | Positive integer | 1 |
| **Apply** | Indicator to apply this configuration | Text | YES/NO | YES |

#### Driver Columns

When using the “wrt\_BAU” option, the scenario does not refer literally to a “BAU” scenario, but to the base scenario that is subsequently defined in the yaml configuration file.

| Column Name | Explanation | Fill Format | Predefined Values | Example |
| --- | --- | --- | --- | --- |
| **Outcome\_ID\_as\_Driver** | ID when an outcome acts as driver, only used when "Driver\_Type" column value is "Metric of interest" or "Intermediary", otherwise left empty, only when "Driver\_U\_ID" column has no values, otherwise left empty. Note that when a driver\_type "Intermediary" is not considered as outcome, no value should be added. | Value from "Outcome\_ID" column of "Outcomes" sheet, with same name | Integer or empty | 2 |
| **Driver\_U\_ID** | Unique driver identifier to avoid duplication, only when "Outcome\_ID\_as\_Driver" column has no values, otherwise left empty. Generally used when driver\_type is "Uncertainty", also when driver\_type is "Intermediary" but not considered as outcome. | Integer or empty | Unique integer or empty | 1 |
| **Driver\_ID** | Sequential driver identifier | Sequential integer | Positive integer sequential starting at 1 | 1 |
| **Driver\_Formula\_Last** | Specifies which period value to use | Text | AVG: average of a period, END: final value of a period, START: initial value of a period | AVG |
| **Driver\_Type** | Driver type classification | Text | Uncertainty: RDM experiment uncertainty, Metric of interest: output metrics of interest, Intermediary: Driver on which metric of interest depends | Intermediary |
| **Driver** | Driver name with suffix indicating analysis type. If "Driver\_Type" has value "Intermediary", name must exactly match names generated in "Outcomes" sheet and for "Uncertainty" value with RDM experiment uncertainty names | Descriptive text | N/A | Benefit AFOLU |
| **Driver\_Actor** | Actor or entity associated with driver (only write the word "Country") | Text | Country | Country |
| **Driver\_Source** | Driver data source | Text | OSeMOSYS-ModelName inputs, OSeMOSYS-ModelName outputs | OSeMOSYS-ModelName outputs |
| **Driver\_Formula\_Numerator** | Numerator formula | Text | Review "Formula Numerator" table | sum\_across |
| **Driver\_Formula\_Denominator** | Denominator formula | Text | none | none |
| **Driver\_Involved\_Sets** | Technologies or fuels involved according to "Driver\_Exact\_Parameters\_Involved" column value | List with format: space ; space | List of technologies/fuels | TRAUTDSL ; TRAUTGSL ; TRAUTELE |
| **Driver\_Supporting\_Sets** | Additional supporting sets, when parameter depends on another set besides TECHNOLOGY OR FUEL, usually if it depends on EMISSION | Text or list with format: space ; space | none or separated list | CO2e |
| **Driver\_Exact\_Parameters\_Involved** | Specific OSeMOSYS and MOMF model parameters. Review available parameters in RDM experiment outputs | List with format: space ; space | Standard OSeMOSYS variables and MOMF specific like Capex2025, VarOpex2025, FixedOpex2025, Externalities2025 | Capex2025 ; VarOpex2025 |
| **Driver\_Tech\_or\_Fuel\_or\_Actor** | Type of modeled element | Text | Tech, Fuel | Tech |
| **Driver\_Column\_Management** | Resulting column management method | Text | none | none |
| **Driver\_Numerator\_Sum\_Sets** | Sets to sum in numerator | If "Outcome\_Variable\_Management" column value is "wrt\_BAU\_excess", value in this column should be "none"; but if value is "Direct" value in this column should be "all" | **all, none** | none |
| **Driver\_Numerator\_Sub\_Sets** | Sets to subtract in numerator | If "Outcome\_Variable\_Management" column value is "wrt\_BAU\_excess", value in this column should be "all"; but if value is "Direct" value in this column should be "none" | **all, none** | all |
| **Driver\_Denominator\_Sum\_Sets** | Sets to sum in denominator | Text | **none** | none |
| **Driver\_Denominator\_Sub\_Sets** | Sets to subtract in denominator | Text | **none** | none |
| **Driver\_Numerator\_Sum\_Params** | Parameters to sum in numerator | If "Outcome\_Variable\_Management" column value is "wrt\_BAU\_excess", value in this column should be "none"; but if value is "Direct" value in this column should be "all" | **all, none** | all |
| **Driver\_Numerator\_Sub\_Params** | Parameters to subtract in numerator | If "Outcome\_Variable\_Management" column value is "wrt\_BAU\_excess", value in this column should be "all"; but if value is "Direct" value in this column should be "none" | **all, none** | all |
| **Driver\_Denominator\_Sum\_Params** | Parameters to sum in denominator | Text | **none** | none |
| **Driver\_Denominator\_Sub\_Params** | Parameters to subtract in denominator | Text | **none** | none |
| **Driver\_Involved\_Scenarios** | Scenarios where driver applies | List with format: space ; space | LTS ; BAU or other defined | LTS ; BAU |
| **Driver\_Variable\_Management** | Management type | Text | Direct: when put only one scenario in "Outcome\_Involved\_Scenarios" column, wrt\_BAU\_excess: with respect SCENARIO | wrt\_BAU\_excess |

#### Outcome Columns

Note that in this "Sequences" sheet several aspects:

1. The "Outcome\_Threshold" column and from "Outcome\_Source" column onwards should only be placed in the first row of a new table, so each time "Table\_ID" column value repeats, no value should be placed in mentioned columns. While for columns: "Outcome\_ID", "Outcome\_Formula\_Last", "Outcome\_Type", "Outcome" and "Outcome\_Actor" the value from the first row of the column should be repeated.
2. Values from these Outcome columns should come from "Outcomes" sheet

| Column Name | Explanation | Fill Format | Predefined Values | Example |
| --- | --- | --- | --- | --- |
| **Outcome\_ID** | Unique outcome identifier | Unique integer | Value must match value from "Outcome" sheet | 1 |
| **Outcome\_Formula\_Last** | Specifies which period value to use | Text | AVG: average of a period, END: final value of a period | AVG |
| **Outcome\_Threshold** | Thresholds for PRIM analysis | List with format: space ; space | High: find predictors of values greater than 75th percentile ; Low: find predictors of values lower than 25th percentile ; Mid: find predictors of values greater than 50th percentile ; Zero: find predictors of values lower than 0 ; Dep: depends on other outcome threshold (High, Low), Intermediary | High ; Low |
| **Outcome\_Type** | Outcome type classification | First use "Metric of interest" which will be the metric of interest for "Outcome" column value, and in following rows use "Intermediary" for variables on which metric of interest depends. | Metric of interest, Intermediary | Metric of interest |
| **Outcome** | Driver name with suffix indicating analysis type. Must exactly match names generated in "Outcomes" sheet | Descriptive text | N/A | Emissions Waste |
| **Outcome\_Actor** | Associated actor or entity (only write the word "Country") | Text | Country | Country |
| **Outcome\_Source** | Data source | Text | OSeMOSYS-ModelName inputs, OSeMOSYS-ModelName outputs | OSeMOSYS-ModelName outputs |
| **Outcome\_Formula\_Numerator** | Numerator formula | Text | Review "Formula Numerator" table | sum\_across |
| **Outcome\_Formula\_Denominator** | Denominator formula | Text | none | none |
| **Outcome\_Involved\_Sets** | Technologies or fuels involved according to "Outcome\_Exact\_Parameters\_Involved" column value | List with format: space ; space | List of technologies/fuels | TRAUTDSL ; TRAUTGSL ; TRAUTELE |
| **Outcome\_Supporting\_Sets** | Additional supporting sets, when parameter depends on another set besides TECHNOLOGY OR FUEL, usually if it depends on EMISSION | Text or list with format: space ; space | none or separated list | CO2e |
| **Outcome\_Exact\_Parameters\_Involved** | Specific OSeMOSYS and MOMF model parameters. Review available parameters in RDM experiment outputs | List with format: space ; space | Variables like Capex2025 ; VarOpex2025 ; FixedOpex2025 ; Externalities2025 | Capex2025 ; VarOpex2025 |
| **Outcome\_Tech\_or\_Fuel\_or\_Actor** | Type of modeled element | Text | Tech, Fuel | Tech |
| **Outcome\_Column\_Management** | Resulting column management | Text | **none** | none |
| **Outcome\_Numerator\_Sum\_Sets** | Sets to sum in numerator | If "Outcome\_Variable\_Management" column value is "wrt\_BAU\_excess", value in this column should be "none"; but if value is "Direct" value in this column should be "all" | **all, none** | none |
| **Outcome\_Numerator\_Sub\_Sets** | Sets to subtract in numerator | If "Outcome\_Variable\_Management" column value is "wrt\_BAU\_excess", value in this column should be "all"; but if value is "Direct" value in this column should be "none" | **all, none** | all |
| **Outcome\_Denominator\_Sum\_Sets** | Sets to sum in denominator | Text | **none** | none |
| **Outcome\_Denominator\_Sub\_Sets** | Sets to subtract in denominator | Text | **none** | none |
| **Outcome\_Numerator\_Sum\_Params** | Parameters to sum in numerator | If "Outcome\_Variable\_Management" column value is "wrt\_BAU\_excess", value in this column should be "none"; but if value is "Direct" value in this column should be "all" | **all, none** | all |
| **Outcome\_Numerator\_Sub\_Params** | Parameters to subtract in numerator | If "Outcome\_Variable\_Management" column value is "wrt\_BAU\_excess", value in this column should be "all"; but if value is "Direct" value in this column should be "none" | **all, none** | all |
| **Outcome\_Denominator\_Sum\_Params** | Parameters to sum in denominator | Text | **none** | none |
| **Outcome\_Denominator\_Sub\_Params** | Parameters to subtract in denominator | Text | **none** | none |
| **Outcome\_Involved\_Scenarios** | Application scenarios | List with format: space ; space | LTS ; BAU or others | LTS ; BAU |
| **Outcome\_Variable\_Management** | Management type | Text | Direct: when put only one scenario in "Outcome\_Involved\_Scenarios" column, wrt\_BAU: with respect BAU | wrt\_BAU |

#### Sheet: Outcomes

Contains only unique outcomes (without repetition per driver). This sheet is a subset of information in Sequences.

| Column Name | Explanation | Fill Format | Predefined Values | Example |
| --- | --- | --- | --- | --- |
| **Outcome\_ID** | Unique outcome identifier | Unique integer | Value must match value from "Outcome" sheet | 1 |
| **Outcome\_Formula\_Last** | Specifies which period value to use | Text | AVG: average of a period, END: final value of a period | AVG |
| **Outcome\_Threshold** | Thresholds for PRIM analysis | List with format: space ; space | High: find predictors of values greater than 75th percentile ; Low: find predictors of values lower than 25th percentile ; Mid: find predictors of values greater than 50th percentile ; Zero: find predictors of values lower than 0 ; Dep: depends on other outcome threshold (High, Low), Intermediary | High ; Low |
| **Outcome\_Type** | Outcome type classification | First use "Metric of interest" which will be the metric of interest for "Outcome" column value, and in following rows use "Intermediary" for variables on which metric of interest depends. | Metric of interest, Intermediary | Metric of interest |
| **Outcome** | Driver name with suffix indicating analysis type. Must exactly match names generated in "Sequences" sheet | Descriptive text | N/A | Emissions Waste |
| **Outcome\_Actor** | Associated actor or entity (only write the word "Country") | Text | Country | Country |
| **Outcome\_Source** | Data source | Text | OSeMOSYS-ModelName inputs, OSeMOSYS-ModelName outputs | OSeMOSYS-ModelName outputs |
| **Outcome\_Formula\_Numerator** | Numerator formula | Text | Review "Formula Numerator" table | sum\_across |
| **Outcome\_Formula\_Denominator** | Denominator formula | Text | none | none |
| **Outcome\_Involved\_Sets** | Technologies or fuels involved according to "Outcome\_Exact\_Parameters\_Involved" column value | List with format: space ; space | List of technologies/fuels | TRAUTDSL ; TRAUTGSL ; TRAUTELE |
| **Outcome\_Supporting\_Sets** | Additional supporting sets, when parameter depends on another set besides TECHNOLOGY OR FUEL, usually if it depends on EMISSION | Text or list with format: space ; space | none or separated list | CO2e |
| **Outcome\_Exact\_Parameters\_Involved** | Specific OSeMOSYS and MOMF model parameters. Review available parameters in RDM experiment outputs | List with format: space ; space | Variables like Capex2025 ; VarOpex2025 ; FixedOpex2025 ; Externalities2025 | Capex2025 ; VarOpex2025 |
| **Outcome\_Tech\_or\_Fuel\_or\_Actor** | Type of modeled element | Text | Tech, Fuel | Tech |
| **Outcome\_Column\_Management** | Resulting column management | Text | **none** | none |
| **Outcome\_Numerator\_Sum\_Sets** | Sets to sum in numerator | If "Outcome\_Variable\_Management" column value is "wrt\_BAU\_excess", value in this column should be "none"; but if value is "Direct" value in this column should be "all" | **all, none** | none |
| **Outcome\_Numerator\_Sub\_Sets** | Sets to subtract in numerator | If "Outcome\_Variable\_Management" column value is "wrt\_BAU\_excess", value in this column should be "all"; but if value is "Direct" value in this column should be "none" | **all, none** | all |
| **Outcome\_Denominator\_Sum\_Sets** | Sets to sum in denominator | Text | **none** | none |
| **Outcome\_Denominator\_Sub\_Sets** | Sets to subtract in denominator | Text | **none** | none |
| **Outcome\_Numerator\_Sum\_Params** | Parameters to sum in numerator | If "Outcome\_Variable\_Management" column value is "wrt\_BAU\_excess", value in this column should be "none"; but if value is "Direct" value in this column should be "all" | **all, none** | all |
| **Outcome\_Numerator\_Sub\_Params** | Parameters to subtract in numerator | If "Outcome\_Variable\_Management" column value is "wrt\_BAU\_excess", value in this column should be "all"; but if value is "Direct" value in this column should be "none" | **all, none** | all |
| **Outcome\_Denominator\_Sum\_Params** | Parameters to sum in denominator | Text | **none** | none |
| **Outcome\_Denominator\_Sub\_Params** | Parameters to subtract in denominator | Text | **none** | none |
| **Outcome\_Involved\_Scenarios** | Application scenarios | List with format: space ; space | LTS ; BAU or others | LTS ; BAU |
| **Outcome\_Variable\_Management** | Management type | Text | Direct: when put only one scenario in "Outcome\_Involved\_Scenarios" column, wrt\_BAU: with respect BAU | wrt\_BAU |

#### Formula Numerator

| Name | Description |
| --- | --- |
| **sum\_across** | Sum all values across specified sets and parameters |
| **average\_across\_rel2023** | Average of all values across specified sets and parameters relative to year 2023. Year value changes according to model base year, the user could change the year |
| **direct** | Direct value without aggregation, typically the last value of period |
| **cumulative** | Cumulative sum across time period |
| **GDP\_growth** | GDP growth rate |
| **share\_H2\_of\_total** | Hydrogen share of total |
| **share\_electric\_of\_total** | Electricity share of total |
| **share\_renewable\_gen** | Non-conventional renewable generation share |
| **special\_elasticity** | Demand elasticity with respect to GDP |
| **special\_FiscalCost** | Net fiscal cost (with and without exemptions) |

### 2. Population.xlsx

**Objective:** Load historical/projected population of study country.

#### Sheet: Sheet1

| Column Name | Explanation | Fill Format | Predefined Values | Example |
| --- | --- | --- | --- | --- |
| **Year** | Population record year | Four-digit integer (YYYY) | 2021-2050 | 2025 |
| **Inhabitants** | Total number of inhabitants for specified year | Absolute integer without separators or decimals | N/A | 2837077 |

**Notes:**

- Include all years of analysis period
- Values should be consistent with official projections

### 3. prim\_files\_creator\_cntrl.xlsx

#### Sheet: match\_exp\_ana

| Column Name | Explanation | Fill Format | Predefined Values | Example |
| --- | --- | --- | --- | --- |
| **exps** | Unique experiment identifier, from experiments available in "experiment\_data" folder | For "1\_Experiment" experiment value is "1\_Experiment" | N/A | 1\_Experiment |
| **exp\_desc** | Detailed experiment description | Descriptive text | N/A | Appends all the elements from multiple models |
| **include\_exp** | Indicator to include experiment in analysis | Text | YES/NO | YES |
| **analyses** | Numerical analysis identifier | Integer | 1, 2, 3... | 1 |
| **analysis\_desc** | Brief analysis description | Brief text | N/A | X and L |
| **include\_ana** | Indicator to include analysis | Text | YES/NO | YES |

#### Sheet: periods

| Column Name | Explanation | Fill Format | Predefined Values | Example |
| --- | --- | --- | --- | --- |
| **period\_list** | Period identifying label. Last two digits of year are used (e.g.: 25 for 2025, 30 for 2030) | Text: initial\_year-final\_year | User defined | 25-30 |
| **year\_initial** | Period start year | Integer (YYYY) | All available model years | 2025 |
| **year\_final** | Period end year | Integer (YYYY) | All available model years | 2030 |

**Note:** Always include an "all" period covering entire time horizon, as the last value

#### Sheet: dtype

| Column Name | Explanation | Fill Format | Predefined Values | Example |
| --- | --- | --- | --- | --- |
| **File** | Data source file type | Text | OSeMOSYS-ModelName inputs, OSeMOSYS-ModelName outputs | OSeMOSYS-ModelName inputs |
| **Column** | Exact column name in source file | Exact text as appears in file | N/A | Technology |
| **Type** | Python data type for column | Text | str, int, float | str |

### 4. Units.xlsx

**Objective:** Document and complete units associated with each driver.

#### Sheet: Units

| Column Name | Explanation | Fill Format | Predefined Values | Example |
| --- | --- | --- | --- | --- |
| **driver\_col** | Driver name with suffix indicating analysis type. Must exactly match names generated in prim\_structure. | Text: Driver\_Outcome\_Variable\_Management where "Driver" is value from "Driver" column in "Sequences" sheet of "prim\_structure" file and "Outcome\_Variable\_Management" is value from "Outcome\_Variable\_Management" column | Must exactly match names in prim\_structure | Benefit EneTra\_Direct |
| **Units** | Measurement unit associated with specified driver | Text: unit abbreviation | MUSD, PJ, GgCO2e, %, GWh, etc. | MUSD |

**Notes:**

- The values for driver\_col must come from prim\_structure.xlsx. For their names, use the value from the “Driver” column + “\_” + “Driver\_Variable\_Management” or “Outcome” + ‘\_’ + “Outcome\_Variable\_Management.”. For example: “Outcome”= “Benefit EneTra”, “Outcome\_Variable\_Management”= “Direct”, so “driver\_col”= “Benefit EneTra\_Direct”
- Values must not be repeated.
- Use standard nomenclature for units

### 5. PRIM\_t3f2.yaml

**Objective:** Configuration file with model parameters.

#### Main structure:

```
# Control files
Prim_files_crea_cntrl: 'prim_files_creator_cntrl.xlsx'

# Excel sheets
Periods: 'periods'
Mat_exp_ana: 'match_exp_ana'
dType: 'dtype'

# Variables and conditions
BAU: 'Scenario1' # Write the name of the base scenario
max_per_batch: 1

ose_inputs: 'OSeMOSYS-Region inputs' # Write the model name with the value of the column "Region" in the sheet "Setup" from the file "Interface_RDM"
ose_oupts: 'OSeMOSYS-Region outputs' # Write the model name with the value of the column "Region" in the sheet "Setup" from the file "Interface_RDM"
```

**Note:** Only change the variables that have the comment beginning with “Write the.”

## Final Reminders

### Important Validations:

1. **Name consistency:** Driver names in Units.xlsx must exactly match those generated by prim\_structure.xlsx
2. **List format:** Always use space + semicolon + space to separate elements (example: "value1 ; value2 ; value3")
3. **Complete periods:** Ensure Population.xlsx covers all years defined in periods
4. **Valid scenarios:** Scenarios in Driver\_Involved\_Scenarios must exist in experiments
5. **Data types:** Verify types in dtype correspond with actual data

### Generated Files:

- `prim_files_creator.pickle` - Processed structure (Intermediate)
- `comp_pfd_*.pickle` - Compiled data per analysis (Intermediate)
- `sd_ana_*_exp_*.csv` - PRIM analysis results (Intermediate)
- `t3f4_predominant_ranges_*.xlsx` - Mapped predominant ranges (Results)

### Verification Order:

1. Verify all Excel files are complete
2. Confirm correct list format (space ; space)
3. Execute t3f1 and verify pickle is generated correctly
4. Review t3f2 logs to confirm experiment processing
5. Validate t3f3 results in CSV file
6. Confirm range mapping in t3f4

## Results Interpretation

After executing the PRIM pipeline, the predominant ranges and key drivers are mapped in the output file located at:

`src\workflow\4_PRIM\t3b_sdiscovery\t3f4_predominant_ranges_a1_e1_Experiment.xlsx`

This file contains the identified uncertainty ranges and their relationship with outcomes. Below is the interpretation guide for each column in the results file:

### Results Columns Interpretation

| Column | Interpretation |
| --- | --- |
| **Outcome\_Type** | Classification of the result type being analyzed: "Desirable" for desirable outcomes (costs and emissions to minimize) or "Risk" for risk zones to monitor. |
| **Metric** | Type of metric used to evaluate the outcome: "Costs" (costs), "Emissions" (emissions), or "All" (multiple combined metrics). |
| **Period** | Temporal range of the analysis expressed in years (e.g., "41-50", "21-30", "31-40"). Defines the evaluation horizon within the project lifecycle. |
| **Driver** | Specific uncertainty factor being analyzed by PRIM (e.g., "Capital Costs ICE Transport", "Variable Costs Crops", "Fuel Demand"). Represents the parameter whose variability is being studied. |
| **Driver\_Type** | Classification of the driver type. Currently only "uncertainty" is available, indicating inherent variability of the parameter. |
| **Min\_Norm** | Minimum normalized value (between 0 and 1) of the uncertainty range identified by PRIM. Lower bound of the region where notable behavior is obtained. |
| **Max\_Norm** | Maximum normalized value (between 0 and 1) of the uncertainty range identified by PRIM. Upper bound of the region. Together with Min\_Norm, they define the PRIM "box". |
| **Case** | Identifier of the scenario or case being analyzed. Groups multiple drivers simultaneously, indicating critical combinations of uncertainties that generate interesting results. |
| **Condition** | Robustness status of the result: "definitive" indicates that the result is conclusive and robust within the identified region. |
| **Tiebreaker** | Additional criterion for resolving trade-offs: "none" (no special criterion) or "high/low average" (when there are conflicts between objectives, specifying whether to maximize or minimize). |

**Understanding the Results:**

- **PRIM Boxes:** The Min\_Norm and Max\_Norm values define "boxes" or regions in the uncertainty space where specific outcomes are most likely to occur.
- **Cases:** Multiple drivers with the same Case number indicate that these uncertainties interact together to produce the observed outcome.
- **Normalized Values:** All values are normalized between 0 and 1, making it easy to compare different uncertainty ranges regardless of their original units.
