# Supplementary material for: OSeMOSYS-RDM: A reproducible workflow for robust decision making with OSeMOSYS models
Source: MethodsX. 2026 Aug 18;17:104102. doi: 10.1016/j.mex.2026.104102 (PMC13543070; doi:10.1016/j.mex.2026.104102)
Supplement: Supplementary file 1 [file mmc1.zip › S4_Perturbation_Methods.docx]

Supplementary Material S4: Parameter Perturbation Methods

This supplementary note provides worked examples for the five parameter perturbation methods supported by OSeMOSYS-RDM. These methods transform sampled values from Latin Hypercube Sampling (LHS) into modified time series that represent plausible alternative futures. Each method addresses different types of uncertainty in energy system parameters.

In OSeMOSYS models, most input parameters are defined as time series spanning the modeling horizon (e.g., 2020–2050). The perturbation methods modify these base case trajectories according to sampled uncertainty values, producing the ensemble of futures required for Robust Decision Making analysis. The choice of method depends on the nature of the uncertainty: whether future values diverge from present knowledge, whether parameters should remain constant, whether trajectories should follow adoption dynamics, whether simple linear interpolation suffices, or whether sub-annual temporal patterns need to vary.

All examples below use a generic time series spanning 2020–2050, with the *Initial_Year_of_Uncertainty* set to 2025. This means values before 2025 remain unchanged, while values from 2025 onward are modified according to the perturbation method. The base case values and LHS samples are illustrative.

# 1 Method 1: Time_Series (Non-linear Interpolation)

The **Time_Series** method modifies the final-year value by a sampled multiplier and interpolates intermediate years while preserving the original trajectory's shape. This is the most commonly used method, appropriate for parameters where current values are well-established but future projections are uncertain—such as technology costs, energy intensities, and demand elasticities.

## 1.1 How it works

The method proceeds in three steps:

1. **Map LHS value to multiplier:** The sampled value (between 0 and 1) is transformed to the parameter's uncertainty range. For example, if the range is [0.8, 1.2] and LHS produces 0.75:

Multiplier = 0.8 + (0.75 × (1.2 − 0.8)) = 1.1

2. **Calculate new final-year value:** Apply the multiplier to the base case's final-year value. If the base case projects 100 units in 2050:

New 2050 value = 100 × 1.1 = 110

3. **Scale year-to-year deltas:** Calculate a slope ratio and apply it to each year-to-year change in the base case, preserving the trajectory's shape.

The slope ratio is computed as:

Slope ratio = (New final value − Anchor value) / (Base final value − Anchor value)

where the anchor value is the value at the Initial_Year_of_Uncertainty. Each year-to-year delta in the base case is then multiplied by this ratio.

## 1.2 Worked example

Consider a technology capital cost that declines from 200 $/kW in 2020 to 100 $/kW in 2050 following a non-linear trajectory (faster decline in early years, slower later). The Initial_Year_of_Uncertainty is 2025, when the cost is 180 $/kW.

Given an LHS sample that produces a multiplier of 1.1:

• New 2050 value = 100 × 1.1 = 110 $/kW

• Slope ratio = (110 − 180) / (100 − 180) = −70 / −80 = 0.875

Each year-to-year change from the base case is scaled by 0.875. The resulting trajectory:

| **Year** | **Base Case ($/kW)** | **Modified ($/kW)** | **Notes** |
| --- | --- | --- | --- |
| 2020 | 200 | 200 | Before uncertainty |
| 2025 | 180 | 180 | Anchor year |
| 2035 | 140 | 145 | Scaled deltas |
| 2050 | 100 | 110 | Target (×1.1) |

*Table 1: Example of Time_Series perturbation preserving trajectory shape*

The key feature is that the modification grows progressively: years near the anchor remain close to the base case, while years approaching the final year diverge toward the target multiplier. The original shape (faster early decline) is preserved.

# 2 Method 2: Constant

The **Constant** method freezes the parameter value at the Initial_Year_of_Uncertainty and maintains that value throughout the remaining modeling horizon. This method is appropriate for parameters where deep uncertainty exists about future trajectories and a constant assumption is analytically useful—such as GDP growth rates or discount rates in sensitivity analysis.

## 2.1 How it works

The procedure is straightforward:

1. Values before Initial_Year_of_Uncertainty remain unchanged

2. The value at Initial_Year_of_Uncertainty becomes the constant value

3. All subsequent years take this constant value

Note that unlike the other methods, Constant does not use the LHS sample to determine a multiplier—it simply holds the trajectory at the anchor year value. The LHS sample may instead determine *which* parameter variation to apply (e.g., selecting between different uncertainty definitions), or the Constant method may be applied unconditionally to explore scenarios where projected changes do not materialize.

## 2.2 Worked example

Consider an energy intensity parameter that in the base case declines from 1.0 MJ/$ in 2020 to 0.6 MJ/$ in 2050, representing anticipated efficiency improvements. Applying the Constant method allows exploration of a scenario where these improvements fail to materialize. With Initial_Year_of_Uncertainty at 2025 (when the value is 0.9 MJ/$):

| **Year** | **Base Case (MJ/$)** | **Constant Method (MJ/$)** |
| --- | --- | --- |
| 2020 | 1.00 | 1.00 |
| 2025 | 0.90 | 0.90 |
| 2035 | 0.75 | 0.90 |
| 2050 | 0.60 | 0.90 |

*Table 2: Example of Constant perturbation freezing at anchor year value*

This method is useful for exploring scenarios where assumed improvements (such as efficiency gains) may not materialize, or for parameters where the base case already uses a constant value and the analyst wishes to preserve that assumption under uncertainty. It can also represent conservative assumptions when there is no reliable basis for projecting future changes.

# 3 Method 3: Linear

The **Linear** method applies simple straight-line interpolation between the anchor year value and a modified final-year value. Unlike Time_Series (which preserves the original trajectory's shape), Linear always produces a straight line regardless of the base case trajectory's shape.

## 3.1 How it works

1. Map the LHS value to a multiplier (same as Time_Series)

2. Calculate the new final-year value by applying the multiplier

3. Linearly interpolate between the anchor year value and the new final-year value

The interpolation formula for each year y between the anchor year and the final year is:

Value(y) = Anchor value + (Final value − Anchor value) × (y − Anchor year) / (Final year − Anchor year)

## 3.2 Worked example

Using the same cost trajectory as Method 1 (200 $/kW in 2020 declining to 100 $/kW in 2050, with anchor at 180 $/kW in 2025), and the same multiplier of 1.1:

• Anchor value (2025) = 180 $/kW

• New final value (2050) = 100 × 1.1 = 110 $/kW

• Linear change per year = (110 − 180) / (2050 − 2025) = −2.8 $/kW/year

| **Year** | **Base Case** | **Time_Series** | **Linear** |
| --- | --- | --- | --- |
| 2020 | 200 | 200 | 200 |
| 2025 | 180 | 180 | 180 |
| 2035 | 140 | 145 | 152 |
| 2050 | 100 | 110 | 110 |

*Table 3: Comparison of Time_Series and Linear methods with identical endpoints*

While both methods reach the same final value, the intermediate trajectories differ. Linear produces uniform year-to-year changes, while Time_Series preserves the base case's pattern of variation. Choose Linear when simplicity is preferred or when the base trajectory's shape is itself highly uncertain.

# 4 Method 4: Logistic

The **Logistic** method generates S-shaped (sigmoid) trajectories characteristic of technology adoption and diffusion processes. Unlike the previous methods that modify existing trajectories, Logistic constructs a new curve from scratch using a logistic function. This method is appropriate for market penetration rates, technology adoption shares, and behavioral transitions.

## 4.1 How it works

The logistic function produces the characteristic S-curve: slow initial growth, rapid acceleration around a midpoint, and gradual saturation approaching a target value. The implementation in OSeMOSYS-RDM uses:

Value(t) = Initial + (Target − Initial) × [1 / (1 + exp(−k × (t − midpoint)))]

where:

**• Initial:** value at the anchor year

**• Target:** final-year value (base case final value × sampled multiplier)

**• k:** growth rate parameter, fixed at 1.0 in the standard implementation

**• midpoint:** center of the S-curve, set automatically to half the number of time steps from the anchor year to the final year

## 4.2 Worked example

Consider technology market share that should transition from near-zero to a target penetration. With anchor year 2025 (initial value = 5%), a target of 80% by 2050, and 26 time steps (2025–2050 inclusive), the midpoint is at step 13 (year 2038):

| **Year** | **Market Share (%)** | **Logistic Factor** |
| --- | --- | --- |
| 2025 | 5.0 | ~0.00 |
| 2030 | 5.0 | ~0.00 |
| 2038 | 42.5 | 0.50 |
| 2045 | 79.9 | ~1.00 |
| 2050 | 80.0 | ~1.00 |

*Table 4: Example of Logistic perturbation generating an S-curve adoption trajectory*

The S-curve reflects realistic adoption dynamics: early adopters represent a small fraction, followed by rapid mainstream adoption around the midpoint, then gradual saturation as the remaining potential market becomes progressively harder to convert. With k=1.0, the transition is relatively sharp, occurring primarily within ±5 years of the midpoint.

# 5 Method 5: Timeslices_Curve

The **Timeslices_Curve** method modifies sub-annual demand profiles (SpecifiedDemandProfile) by selecting from a set of predefined load shape curves. Unlike the previous four methods that modify annual time series values, this method operates on the temporal disaggregation of demand across time slices within each year.

## 5.1 How it works

The method uses the LHS sample to select among predefined alternative demand profiles:

1. A CSV file defines multiple demand shape curves (columns), each representing a plausible sub-annual demand pattern

2. The uncertainty range [Min_Value, Max_Value] from the Uncertainty_Table is divided into equal intervals, one per available curve

3. The LHS sample determines which interval (and thus which curve) is selected

4. The selected curve's values replace the SpecifiedDemandProfile for all years in the modeling horizon

## 5.2 Worked example

Consider residential electricity demand with uncertainty in the sub-annual consumption pattern. Three alternative load shapes are defined in a CSV file:

**• Curve 1 ("Flat"):** Relatively uniform consumption throughout the year

**• Curve 2 ("Summer Peak"):** Higher consumption during summer months (cooling-dominated)

**• Curve 3 ("Winter Peak"):** Higher consumption during winter months (heating-dominated)

With Min_Value=0 and Max_Value=1, the range is divided into three equal intervals.

*Table 5: Example of Timeslices_Curve selection based on LHS sample*

| **LHS Sample Range** | **Selected Curve** | **Demand Pattern** |
| --- | --- | --- |
| 0.00 – 0.33 | Curve 1 | Flat |
| 0.33 – 0.67 | Curve 2 | Summer Peak |
| 0.67 – 1.00 | Curve 3 | Winter Peak |

If the LHS sample produces 0.55, this falls in the second interval, selecting the "Summer Peak" curve. The selected profile values then replace the SpecifiedDemandProfile across all time slices for the specified commodity.

This method is particularly useful for exploring uncertainty in demand patterns driven by climate variability, behavioral changes, or the adoption of end-use technologies (such as air conditioning or electric vehicles) that alter when energy is consumed.

# 6 Summary

The choice of perturbation method should reflect the nature of uncertainty for each parameter:

*Table 6: Summary of perturbation methods and their appropriate applications*

| **Method** | **Use When** | **Effect on Trajectory** |
| --- | --- | --- |
| **Time_Series** | Current values known, future uncertain; trajectory shape meaningful | Modifies final value, preserves trajectory shape |
| **Constant** | No reliable basis for projecting future changes | Freezes at anchor year value throughout horizon |
| **Linear** | Trajectory shape uncertain; simplicity preferred | Straight-line interpolation to modified final value |
| **Logistic** | Adoption/penetration processes with S-curve dynamics | Generates S-curve from initial to target value |
| **Timeslices_ Curve** | Sub-annual demand patterns uncertain; alternative load shapes available | Selects from predefined temporal profiles |
