# Supplementary material for: OSeMOSYS-RDM: A reproducible workflow for robust decision making with OSeMOSYS models
Source: MethodsX. 2026 Aug 18;17:104102. doi: 10.1016/j.mex.2026.104102 (PMC13543070; doi:10.1016/j.mex.2026.104102)
Supplement: Supplementary file 1 [file mmc1.zip › S5_Concurrency analysis.docx]

Supplementary material S5: Concurrency analysis example

This supplementary note clarifies when driver conditions identified by the Patient Rule Induction Method (PRIM) implementation in OSeMOSYS-RDM. must be interpreted as concurrent (all conditions met simultaneously) versus independent. This distinction is essential for interpreting scenario discovery results and for conducting trade-off or synergy analyses across multiple metrics.

# 1 What is a PRIM box?

A PRIM box defines a region in the input space where a specific outcome of interest is concentrated. Geometrically, a box is a hyper-rectangle defined by value ranges on one or more input variables (drivers). For a two-dimensional example, consider drivers x_1_ and x_2_. A box B might be defined as:

*x* ∈ B if and only if *a* ≤ x_1_ ≤ *b* AND *c* ≤ x_2_ ≤ *d*

This means that a future belongs to box B only if *both* conditions are satisfied simultaneously. Supplementary Figure 6.1 illustrates this concept visually, adapted from Nassih and Berrado (2024).

**Supplementary Figure S6.1.** Two-dimensional representation of a PRIM box. A gray rectangle B shows the region where x_1_ ∈ [a, b] AND x_2_ ∈ [c, d]. Points inside the box represent futures with the outcome of interest; points outside do not meet all conditions that explain an outcome of interest.

Although this example uses two dimensions for clarity, PRIM boxes applied in larger models are often defined by more drivers. For instance, in the Costa Rica transport decarbonization study (main text), PRIM analyzed 28 candidate input variables, with selected boxes sometimes involving about 10 concurrent driver conditions (Victor-Gallardo, L.F., 2022).

# 2 The concurrency rule within a single box

Within any single PRIM box, all identified driver conditions are strictly concurrent. This is not a methodological choice but a mathematical consequence of how PRIM defines boxes. If PRIM identifies three drivers for a given outcome combination (e.g., desirable capital costs), then all three conditions must be met simultaneously for a future to fall within that box and thus be associated with the outcome.

Consider a concrete example. Suppose PRIM identifies the following box for *desirable system costs* (costs in the bottom 25th percentile):

| Driver | Original range | Normalized range |
| --- | --- | --- |
| BEV battery cost | [80, 115] USD/kWh | Normalized: [0.20, 0.55] |
| Fuel demand growth | [0.85, 1.05] | Normalized: [0.35, 0.75] |
| Infrastructure cost | [0, 95] index | Normalized: [0.00, 0.45] |

A future achieves desirable system costs only if *all three* conditions hold: battery costs are moderately low, fuel demand growth is moderate, *and* infrastructure costs remain below 45% of their maximum value explored. Meeting only two of three conditions does not guarantee the desirable outcome.

# 3 Independence across different outcome boxes

When PRIM is applied to multiple outcome combinations (e.g., desirable costs, desirable emissions, risk of high costs, risk of high emissions), each analysis produces a *separate and independent* box. These boxes are not automatically concurrent with each other. Note each outcome is either desirable or risk, with an associated metric.

The key insight is that a driver appearing in multiple boxes may have *different* or even *opposing* ranges associated with each outcome. This creates three possible relationships when comparing boxes:

- **Synergy:** Driver ranges overlap substantially or point in the same direction across metrics. Achieving one outcome does not preclude the other.
- **Trade-off:** Driver ranges conflict or point in opposite directions. Achieving one outcome may preclude or hinder the other.
- **Independence:** Different drivers govern different outcomes, with no shared variables creating direct conflict or alignment.

# 4 Illustrative examples

The following examples demonstrate how to interpret driver conditions across two outcome boxes. All ranges are normalized to [0, 100] for comparability.

**Example 1: Synergy**

| Driver | BOXES  (outcome combinations) | |
| --- | --- | --- |
|  | Desirable Costs | Desirable Emissions |
| BEV battery cost | [20, 55] | [15, 50] |
| Electrification mandate | [60, 100] | [70, 100] |

Both outcomes favor low battery costs and high electrification mandates. The ranges overlap at BEV cost ∈ [20, 50] and electrification ∈ [70, 100]. Within this overlapping region, futures can achieve *both* desirable costs and desirable emissions simultaneously. This is a synergy: policies that drive outcomes toward one box also drive toward the other.

**Example 2: Trade-off**

| Driver | BOXES  (outcome combinations) | |
| --- | --- | --- |
|  | Desirable Costs | Desirable Emissions |
| Electrification mandate | [0, 40] | [70, 100] |
| Biofuel subsidy | [0, 30] | [50, 90] |

Here the ranges *do not overlap*. Desirable costs require low electrification mandates and low biofuel subsidies, while desirable emissions require high values of both. No future can simultaneously satisfy both boxes. This is a trade-off: pursuing cost reduction conflicts directly with emissions reduction.

**Example 3: Partial overlap (mixed synergies and trade-offs)**

| Driver | BOXES  (outcome combinations) | |
| --- | --- | --- |
|  | Desirable Costs | Desirable Emissions |
| BEV battery cost | [20, 55] | [10, 45] |
| Electrification mandate | [0, 50] | [60, 100] |
| Fuel price | — (not a driver) | [55, 85] |

BEV battery cost shows a synergy (overlap at [20, 45]), while electrification mandate shows a trade-off (no overlap). Fuel price affects only emissions, creating independence on that dimension. This mixed pattern is common when applied to large models and relatively complicated experiments, thus requiring nuanced interpretation: decision-makers can rely on battery cost improvements to support both goals, but must make explicit choices about electrification policy knowing it involves a trade-off.

## 4.1 When to conduct cross-box analysis

Cross-box comparison (synergy and trade-off analysis) is warranted when:

(a) Multiple metrics are policy-relevant, and decision-makers need to understand whether improving one outcome helps or hinders another.

(b) The same drivers appear across multiple boxes, suggesting potential interactions.

(c) Robust strategies are sought to perform well across multiple criteria, not just one.

Cross box analysis is *not* needed when only a single metric (outcome) is of interest, or when boxes involve entirely different drivers with no overlap in the input variables are considered.

# 5 Summary

The key distinction can be summarized as follows:

- **Within a box:** All driver conditions are mandatory and concurrent. A future must satisfy every identified range to belong to that box.
- **Across boxes:** Results are independent. Ranges for the same driver may differ across metrics, requiring explicit comparison to identify synergies, trade-offs, or independence.

Users should interpret each outcome result on its own terms before comparing across results. The workflow described in the main text supports this by producing separate driver-range tables for each outcome combination, enabling systematic cross-comparison when desired.

# 6 References

Nassih, R., & Berrado, A. (2024). A Random PRIM Based Algorithm for Interpretable Classification and Advanced Subgroup Discovery. *Algorithms*, *17*(12), 565. https://doi.org/10.3390/a17120565

Victor-Gallardo, L.F. (2022). *Robust energy system planning for decarbonization under technological uncertainty: From transport electrification to power system investments*. Repositorio Institucional Kérwá (Universidad de Costa Rica). [https://www.kerwa.ucr.ac.cr/items/fa1f4673-2854-4d2b-b43f-fb8bc5d98a06](https://www.kerwa.ucr.ac.cr/items/fa1f4673-2854-4d2b-b43f-fb8bc5d98a06?utm_source=chatgpt.com) (Handle: https://hdl.handle.net/10669/87273).
