# Supplementary material for: OSeMOSYS-RDM: A reproducible workflow for robust decision making with OSeMOSYS models
Source: MethodsX. 2026 Aug 18;17:104102. doi: 10.1016/j.mex.2026.104102 (PMC13543070; doi:10.1016/j.mex.2026.104102)
Supplement: Supplementary file 1 [file mmc1.zip › S6_OSeMOSYS-RDM-Example-Experiment.docx]

Supplementary Material S6: OSeMOSYS-RDM Example: Experiment Component

# 1 Country Context: Uganda

## 1.1 Overview

Uganda is a landlocked country in East Africa with an area of about 241,037 km² (UN Uganda, 2022). It is largely tropical, influenced by the Intertropical Convergence Zone, and experiences two main seasons: March–May and October–December. The population is among the youngest in the world, with more than 70 percent under the age of 30 and around three-quarters living in rural areas (UN Uganda, 2022).

The country’s economy is driven by agriculture, which employs over 70 percent of the population and remains highly dependent on rainfall (FAO, 2024; WBG, 2025). GDP growth averaged about 6 percent in 2024 (UN Uganda, 2025), supported by recovery in services, industry, and agriculture and by expectations of oil production beginning in 2025. However, inequality, regional disparities, and high youth unemployment persist—poverty rates exceed 60 percent in Karamoja and Acholi compared to less than 10 percent in Kampala (UN Uganda, 2025; Vaughan, 2025). The OSeMOSYS model for Uganda is under development and the purpose of documenting this experimentation analysis is to demonstrate the application of the OSeMOSYS-RDM workflow.

## 1.2 Sustainable Development Framework

Uganda’s long-term ambition is articulated in *Vision 2040*, which aims to transform the country from a “peasant society” into a modern and prosperous nation within 30 years. The strategy is implemented through a sequence of 10-year and 5-year development plans (UN Uganda, 2022).

The Third National Development Plan (NDP III, 2020/21–2024/25) seeks to raise household incomes and improve quality of life through resource-led industrialization and investments in agriculture, tourism, oil and gas, and minerals. It promotes a programmatic approach to service delivery, stronger state participation in productive investment, and digitalization to improve efficiency (UN Uganda, 2022). The upcoming NDP IV (2026–2030) sets out a “ten-fold growth” agenda anchored in inclusive economic transformation, climate resilience, and delivery through the Parish Development Model (PDM) (LDPG, n.d.; MOFPED, 225 C.E.).

Uganda has integrated the Sustainable Development Goals (SDGs) into its planning frameworks. Progress has been moderate in agriculture, health, energy, and industry but slower in reducing poverty and inequality, ensuring gender equality, and achieving climate-action-related goals (UN Uganda, 2022).

## 1.3 Climate Change Profile

Both temperature and rainfall are projected to increase across Uganda, although rainfall patterns remain uncertain (WBG, 2025). The country faces recurrent droughts, floods, and landslides—such as the 2022 Kisoro landslides and prolonged droughts in Karamoja, which led to severe food insecurity affecting over 40 percent of households.

Agriculture, forestry, and other land use (AFOLU) are the main greenhouse-gas sources, contributing more than 80 percent of emissions, followed by transport, energy (MWE, 2025). Under the Business-As-Usual (BAU) trajectory, national emissions are projected to rise from 90.1 MtCO₂e (2015) to 148.8 MtCO₂e (2030) and 235.7 MtCO₂e (2050) (MWE, 2022).

Vulnerability is greatest in climate-sensitive regions such as Karamoja, Teso, and the western highlands (GIZ, 2021). Women are disproportionately affected due to unequal access to land, finance, and decision-making power (UN Uganda, 2022).

## 1.4 Policy and Institutional Framework

Uganda’s Updated Nationally Determined Contribution (2022) commits to an economy-wide emissions reduction of 24.7 percent below BAU by 2030^[1]^. Adaptation remains the country’s top climate priority, covering 13 sectors including agriculture, forestry, energy, health, ecosystems, water and sanitation, transport, manufacturing, industry, cities, disaster risk reduction, tourism, and education (MWE, 2022).

Uganda’s National Climate Change Act (2021) operationalizes the UNFCCC, Kyoto Protocol, and Paris Agreement, defining roles for the Ministry of Water and Environment’s Climate Change Department, the National Climate Change Advisory Committee, and subnational climate committees (MWE, 2022).

The 2022’s NDC and the Long-Term Low Emissions Development Strategy (LTS) link short-term actions with long term vision, focusing on low-carbon growth and resilience building. Complementary frameworks include the National Green Growth Strategy (2017–2030) and the 10-Year Environment Restoration Plan (MWE, 2022).

## 1.5 A landscape for energy planning

Uganda’s pursuit of inclusive growth under Vision 2040, together with its exposure to climate risks and its evolving climate-policy architecture, creates a complex but analytically rich landscape for resilience planning. The multisectoral OSeMOSYS–RDM modeling exercise draws on this national context to test development pathways that can withstand uncertainty, support national objectives, and advance climate-resilient sustainable development.

# 2 Experiment component configuration

## 2.1 Setup Configuration Analysis

The Setup sheet defines the fundamental parameters governing the RDM experiment execution. The configuration establishes a controlled experimental environment with the following key specifications:

The experiment is configured to generate 20 different future scenarios (Number_of_Runs = 20), each representing a unique combination of uncertainty parameter values. The experimental design targets the "Experiment" scenario type with a unique identifier (Experiment_ID = 1) to distinguish this particular analysis from other potential RDM studies.

Computational efficiency is optimized through parallel processing capabilities, with the system configured to execute 10 simultaneous model runs (Parallel_Use = 10). This parallelization approach significantly reduces the overall computation time while maintaining system stability within the hardware specifications of the target machine.

The model structure incorporates 8 timeslices (Timeslices_model = 8), providing temporal granularity for the energy system optimization. The experimental protocol includes execution of both baseline scenarios (Run_Base_Future = Yes) and the full RDM analysis (Run_RDM = Yes), ensuring comprehensive coverage of both reference conditions and uncertainty exploration.

The optimization engine employs the CBC (Coin-or Branch and Cut) solver, with model calculations based on version 5.3 of the OSeMOSYS framework (OSeMOSYS_Model_Name = model.v.5.3.txt). The uncertainty analysis commences from the year 2025 (Initial_Year_of_Uncertainty = 2025), establishing the temporal starting point for parameter variations.

## 2.2 Uncertainty Table Analysis

The Uncertainty_Table sheet defines 15 distinct uncertainties across multiple technology and sector categories, each characterized by specific mathematical treatments and parameter ranges. The uncertainty structure demonstrates systematic coverage of key model components affecting energy system behavior and outcomes.

## 2.3 Uncertainty Categories and Distribution

The uncertainty parameters span seven primary categories reflect different aspects of energy system costs and performance. Collection rate uncertainties address waste management system efficiency, with variations ranging from 0.6 to 1.0 for liquid waste collection and 0.8 to 1.0 for solid waste collection systems. These parameters directly influence waste-to-energy conversion pathways and disposal requirements.

Output Activity Ratio uncertainties target agricultural land use technologies, with parameter variations between 0.8 and 1.2, affecting crop production efficiency across multiple agricultural systems including plantain, maize, cassava, beans, coffee, ground nuts, potatoes, sunflower, and other crop categories. These variations capture potential changes in agricultural productivity under different climatic and management conditions.

Agricultural cost uncertainties reflect economic variability in crop production systems. Variable costs for crop technologies range from 0.9 to 1.3, while fixed costs vary between 0.8 to 1.4, reflecting potential economic uncertainties in agricultural operations and maintenance requirements.

Electric vehicle cost uncertainties address transportation electrification economics. Capital costs for electric vehicle technologies vary between 0.7 and 1.4, while fixed costs range from 0.9 to 1.2, capturing potential changes in electric vehicle acquisition and maintenance costs across different vehicle categories.

Internal Combustion Engine (ICE) vehicle cost uncertainties complement the electric vehicle analysis. Capital costs for ICE vehicles range from 0.9 to 1.2, while fixed costs vary between 0.9 to 1.1, reflecting potential economic changes in conventional vehicle technologies and their operational requirements.

Demand growth uncertainties incorporate systematic variations in energy consumption patterns, with parameter ranges from 0.9 to 1.2. These uncertainties address potential changes in energy demand across different sectors and end-use categories, reflecting economic growth, population changes, and behavioral modifications.

Fuel import cost uncertainties address economic variability in energy supply chains. Variable costs for biomass imports range from 0.8 to 1.4, while fuel import costs vary between 0.7 to 1.5, capturing potential changes in international commodity prices and supply chain logistics affecting energy system economics.

Input Activity Ratio uncertainties, ranging from 0.9 to 1.2, affect fuel conversion efficiencies and resource utilization rates across various energy transformation processes.

Industrial production cost uncertainties encompass two distinct technology categories within steel and cement sectors. Capital costs for steel direct electric arc furnace with hydrogen technologies and cement industry kilns with carbon capture and storage (CCS) range from 0.8 to 1.5. Alternatively, capital costs for steel direct electric arc furnace with natural gas technologies and cement industry kilns without CCS vary between 0.9 to 1.3, reflecting different technological approaches and their associated investment requirements.

## 2.4 Mathematical Treatment and Parameter Manipulation

All uncertainty parameters employ "Time_Series" mathematical treatment with "Final_Value" exploration methodology. This approach modifies the final values of time series data by multiplying them with explored parameter values, which are randomly defined using Latin Hypercube Sampling (LHS) method within the specified ranges. The modified parameters are then interpolated to generate complete temporal profiles and subsequently reassigned to the OSeMOSYS model structure for each experimental run.

The uncertainty analysis targets specific OSeMOSYS parameters including UDCMultiplierActivity, OutputActivityRatio, VariableCost, FixedCost, CapitalCost, SpecifiedAnnualDemand, and InputActivityRatio. These parameters directly influence model optimization behavior and solution characteristics across different future scenarios.

## 2.5 Technology and Fuel Coverage

The uncertainty analysis encompasses extensive technology and fuel coverage across multiple sectors. Agricultural technologies include land-use systems for various crops (LNDPLAHR, LNDMAIHR, LNDCASHR, etc.), while transportation uncertainties focus primarily on electric vehicle technologies (TRAELCCARCUR, TRAELCCARNEW, TRAELCMCYCUR, etc.). Power generation uncertainties address renewable and conventional generation technologies, including wind, solar, hydroelectric, thermal, and biomass systems.

The comprehensive scope of technology coverage ensures that key uncertainties affecting system-wide energy flows, costs, and emissions are systematically explored within the experimental framework, providing robust analysis capabilities for strategic decision-making under uncertainty.

# 3 Results

The following section summarizes the principal findings and interpretations derived from the applying an experiment to an Uganda model. As the modeling exercise employed normalized outputs with unspecified units, the insights discussed here should be understood in relative rather than absolute terms, focusing on comparative trends and systemic behavior rather than quantitative magnitudes.

## 3.1 Modelling and RDM

Figure 1 illustrates how the Land Use and Mining sectors evolve over time under multiple future scenarios in the RDM experiment.

- **Land Use sector:**

After excluding high-value technologies to focus on those showing inter-future variability, the Land Use sector displays a gradual decline in total capacity from about 10 000 units in 2021 to between 7 000 and 9 000 units by 2050. Its overall activity follows a similar downward trend, modeling a modest contraction of land-based technologies over the long term.

- **Mining sector:**

In contrast, the Mining sector shows steady growth throughout the projection period. Both its capacity and activity rise consistently, reaching around 3 000 units by 2050, with minimal divergence across futures, indicating relative stability and low sensitivity to uncertainty.

Overall, Figure 1 highlights diverging dynamics between the two sectors: while land-use technologies decline slightly under most futures, mining technologies expand steadily and robustly across the 21 simulated scenarios.

Figure 1: Capacity Annual and Total Technology Annual Activity by sector

Figure 2 complements the previous analysis by translating the physical evolution of technologies into their financial expression. It compares the behavior of Land Use and Transport sectors through three key cost dimensions — Capital Investment, Annual Fixed Operating Cost, and Annual Variable Operating Cost — across the 21 simulated futures.

In the Land Use sector, investment patterns reveal occasional cost spikes reaching up to 3,500 units, followed by periods of decline and stabilization. These peaks likely represent renewal cycles or short-term responses to productivity shocks. Over time, both fixed and variable operating costs decrease from approximately 12,000 to 7,000–9,000 units and 18,000 to 11,000–16,000 units, respectively, indicating a gradual contraction of agricultural and land-based activities. The broad uncertainty around these trajectories reflect the sector’s sensitivity to fluctuating yields, input costs, and climate-related pressures. Together, these results signal growing financial volatility in land systems, consistent with the physical decline observed in Figure 1.

Figure 2 Capital Investment, Annual Fixed Operating Cost and Annual Variable Operating Cost by sector

The Transport sector, in contrast, exhibits a steady and capital-intensive expansion. Investment rises sharply throughout the projection period, reaching to 20,000 units by 2050, while fixed operating costs increase in parallel to about 13,000–14,500 units. Variable operating costs also grow gradually, reflecting continuous utilization and network expansion. This pattern mirrors Uganda’s ongoing prioritization of infrastructure and mobility within its national development agenda, which may include road and logistics investments, urban transport upgrades, and emerging interest in electric vehicles.

Overall, Figure 2 underscores a structural divergence between the two sectors. While Land Use systems face declining efficiency and rising uncertainty, Transport becomes an increasingly dominant and capital-driven component of Uganda’s long-term development pathway.

## 3.2 Interpreting system vulnerabilities and drivers of robustness (PRIM results)

The final phase of the analysis applied the Patient Rule Induction Method (PRIM) to identify the conditions under which Uganda’s multisectoral system performs robustly or becomes vulnerable under uncertainty^[2]^. Whereas the previous figures illustrated how sectors evolve in scale, cost, and efficiency, the PRIM results reveal why those trajectories emerge—highlighting the parameters that most strongly influence long-term robustness.

Four key insights stand out:

- **Electric transport is essential.** Across all modeled futures, investment in electric mobility remains a foundational condition for achieving both cost efficiency and emissions reduction. The consistently high contribution of this variable confirms that electrified transport is a structural pillar of a low-carbon pathway.
- **Industry emissions are the swing factor.** The analysis shows that timing and scale of industrial investment determine whether national emissions targets are met without excessive cost.
- **Cost–emission.** The model indicates that early mitigation concurs with low-cost outcomes.
- **Demand management gains prominence over time.** The increasing appearance of residential and fuel-demand constraints in the later years points to a plausible rising importance of efficiency measures and behavioral shifts.

Together, these findings provide a strategic perspective for Uganda’s transition planning. They suggest that early and coordinated investments in electric transport, clean industry, and demand-side management can deliver both economic resilience and climate alignment, supporting the national goals set out in Vision 2040, the NDP IV, and the updated NDC (2022).

# 4 References

FAO. (2024). Uganda at a glance [Org]. FAO in Uganda. https://www.fao.org/uganda/our-office/uganda-at-a-glance/en

GIZ. (2021). Climate Risk Profile: Ugande. Adaptation Community. https://www.adaptationcommunity.net/wp-content/uploads/2021/02/GIZ_Climate-risk-profile-Uganda_EN_final.pdf

LDPG. (n.d.). Vision 2040 and National Development Plans. https://www.ldpg.or.ug/frameworks/vision-2040-and-national-development-plans/

MOFPED. (225 C.E., June). 10-fold Growth Strategy. GoUg. https://create.finance.go.ug/sites/default/files/2025-06/TENFOLD%20GROWTH%20STRATEGY.pdf

MWE. (2022). Updated Nationally Determined Contribution (NDC). UNFCCC. https://unfccc.int/sites/default/files/NDC/2022-09/Updated%20NDC%20_Uganda_2022%20Final.pdf

MWE. (2025). Uganda’s Second Biennial Update Report to the United Nations Framework Convention on Climate Change.

UN Uganda. (2022). Common Country Analysis (2022 Update). https://uganda.un.org/en/228935-common-country-analysis-uganda-2022-update

UN Uganda. (2025). Annual Results Report 2024. UN Uganda. https://uganda.un.org/en/297765-un-uganda-annual-results-report-2024

Vaughan, K. N. (2025, October). Poverty & Equity Brief: Uganda. WBG. https://documents1.worldbank.org/curated/en/099523204222530354/pdf/IDU-30f95e03-7d91-400b-a4a5-d68c284d6f97.pdf

WBG. (2025). Uganda: Country Climate and Development Report. WBG. https://openknowledge.worldbank.org/server/api/core/bitstreams/35aaaaec-8886-48ca-b5bb-9f133740e57f/content

1. From the 24.7%, 5.9 percent is unconditional, and 18.8 percent is conditional, depending on international support for finance, capacity-building, and technology.
2. *Since the current excercise explored a relatively small ensemble of 20 futures to demonstrate how the RDM–PRIM framework can support decision-making under uncertainty, these results should be interpreted as indicative patterns only. Larger and more targeted experiments could refine these insights, better capture cross-sectoral interactions, and strengthen the statistical robustness of the drawn insightspresented at this stage.*
