# Supplementary material for: OSeMOSYS-RDM: A reproducible workflow for robust decision making with OSeMOSYS models
Source: MethodsX. 2026 Aug 18;17:104102. doi: 10.1016/j.mex.2026.104102 (PMC13543070; doi:10.1016/j.mex.2026.104102)
Supplement: Supplementary file 1 [file mmc1.zip › S7_OSeMOSYS-RDM-Example-PRIM.docx]

Supplementary Material S7: PRIM summary report

## 1. Background

To identify the main factors that explain when the modeled system performs robustly or fails to meet the desired outcomes^[1]^, we applied the Patient Rule Induction Method (PRIM). PRIM is a data-mining algorithm commonly used for scenario discovery within Robust Decision Making (RDM) frameworks. It helps uncover combinations of uncertain factors (hereafter drivers) that are systematically associated with specific types of outcomes across a large ensemble of simulated futures. Before presenting the results of the PRIM analysis for the Ugandan OSeMOSYS model–RDM exercise, the current section introduces key concepts and terminology to help interpret the findings presented in the Results section.

### 1.1. Overview

Scenario discovery provides a bridge between large-scale computational experiments and policy interpretation. Rather than seeking to predict single outcomes, it identifies conditions (i.e., ranges of uncertain parameters) under which a given strategy performs particularly well or poorly. PRIM aids this process by iteratively exploring the uncertainty space to locate “boxes” (subsets of futures) where the modeled outcomes exhibit similar characteristics, such as high costs, low emissions, or economic gains. This approach helps analysts understand why certain futures are favorable or unfavorable for a strategy, offering insight into its vulnerabilities and opportunities under deep uncertainty.

### 1.2. Input Data and Outcomes of interest

The analysis used results from the OSeMOSYS experiments, which generated 20 alternative futures representing different combinations of uncertain variables related to capital costs investments, operational costs and demand behaviors (see Supplementary Material S6). Each future corresponds to one simulation run, producing a set of performance metrics (e.g., costs, emissions and the combined metric of both). The PRIM algorithm takes this dataset (uncertain inputs paired with outcomes) and searches for combinations of input conditions that consistently lead to particular types of results.

For this exercise, we defined 4 conditions of interest^[2]^ representing distinct policy-relevant outcomes:

Table 1: Conditions representing outcomes of interest

|  | Conditions of interest (combination of metrics w/ specific values) |
| --- | --- |
| Desirable outcome | Low emissions, low costs |
| Risk outcome | High emissions, high costs |

Each performance metric takes one value for every simulated future in the dataset. To define the “high” and “low” outcome conditions, we use percentile thresholds based on the full distribution of results across all simulated futures. Specifically, “high” outcomes correspond to the [upper 25%] (top quartile) of values for a given metric, while “low” outcomes correspond to the [lower 25%] (bottom quartile).

### 1.3. The PRIM steps

Figure 1 provides a simplified overview of the main steps of the PRIM algorithm (Friedman & Fisher, 1999):

1. Initiatilization

Start with complete set of futures simulated futures.

2. Peeling

Iteratively remove small portions of cases. This process “peels away” less relevant regions to isolate those where the condition of interest is concentrated (the boxes of interest.

3. Pasting

After peeling, the algorithm may slightly expand (“paste”) the boundaries of the box to improve generality.

4. Box evaluation

Each box is characterized and assesed according to coverage, density and interpretability

5. Selection

The most relevant boxes are selected aby balancing coverage, density and interpretability.

Results are interpreted

Figure 1: PRIM algorithmic procedure

Once potential “boxes” or regions of interest have been identified, each is assessed through coverage, density, and interpretability (4^th^ step, Figure 1), namely, the three complementary criteria that balance precision and generality.

- Coverage measures how much of the data (or how many cases) are included in a box. A box with high coverage represents a large portion of the possible futures or scenarios, indicating that the identified pattern is broadly applicable.
- Density measures how strongly the condition of interest is concentrated within the box. It is the average value of the target variable inside the box compared to its global average. High density means that the region contains a high concentration of outcomes of interest (i.e. low emissions cases) even if it covers only a small part of the data (small coverage) ^[3]^.
- Interpretability refers to how easily the defining conditions of a box can be expressed and understood. A box defined by only a few, clearly stated variables (drivers) and threshold values (the ranges in the box) is easier to communicate and act upon than one that involves many complex conditions.

In practice, PRIM operationalizes these criteria differently based on box complexity. For simple boxes (≤2 drivers), it maximizes the average of coverage and density. For complex boxes (>2 drivers), it employs a two-stage strategy: first filtering for dimensions that achieve at least 70% coverage, then selecting the most restrictive box that maintains this threshold. This ensures that multi-dimensional patterns remain broadly applicable and interpretable rather than describing rare, overly specific combinations. The selected box is ultimately characterized by its coverage (proportion of relevant cases captured), density (concentration ratio of the target condition), and a composite merit score that balances both metrics.

### 1.4. Interpretation of results

The PRIM algorithm yields a set of decision-relevant conditions, each defining ranges of uncertain variables that correspond to specific outcomes. The results in the upcoming section are presented in the form of matrices, one for each outcome type and performance metric (four in total). The conditions shown in each matrix (the variable ranges) can be interpreted as vulnerabilities (combinations of uncertainties under which the system performs poorly) or opportunities (combinations under which it performs well). Among the different boxes identified, priority is often given to those that capture clear and consistent relationships between input drivers and outcomes. Boxes that include a greater number of fixed parameters (variables with constrained ranges) tend to be more specific, providing richer insight into the combinations of uncertainties that drive system behavior. While these boxes may cover a smaller share of futures, they are often the most informative for guiding decisions and improving the robustness of policy strategies.

It is important to note that the variable ranges in these matrices should not be interpreted as fixed thresholds. Each matrix isolates a subset of futures characterized by distinct combinations of uncertainty conditions, meaning that the same variable may contribute to positive outcomes under one context and negative outcomes under another. Apparent contradictions between desirable and risk ranges reflect the non-linear and context-dependent nature of system behavior. These overlaps are analytically valuable, as they reveal where trade-offs or sensitivities emerge across objectives such as costs and emissions, highlighting the boundaries of robust performance under deep uncertainty.

Ultimately, PRIM helps translate the complex relationships in the model ensemble into a small set of understandable “if–then” statements, clarifying when and why a policy performs well or fails. When interpreted across matrices, these insights help identify robust zones (parameter spaces that perform acceptably across multiple objectives) rather than single-point optima. In doing so, the methodology can help inform national climate strategies by highlighting the uncertainty conditions that most affect its success.

## 2. Results

The tables below reveal which drivers (uncertainties and levers) are most critical for achieving either low emissions and low costs (desirable outcomes, as in Table 2 and Table 4) or which ones lead to scenarios with high emissions and high costs (risk outcomes, as in Table 3 and Table 5). The colored ranges are illustrated in Figure 2:

Figure 2: Color coding of range values

Note on empty cells: Drivers that PRIM did not identify as significant for the outcome in question

### 2.1. Emissions

The PRIM analysis reveals specific driver ranges that lead to specific outcomes (low or high emissions) across the three analysis periods. Critically, these ranges represent concurrent conditions, meaning all identified drivers within each period must be satisfied simultaneously to achieve the futures identified by the algorithm.

Table 2: Drivers of the desirable outcome for Emissions (low)

| **Drivers** | | **Desirable outcomes** | | | | | |
| --- | --- | --- | --- | --- | --- | --- | --- |
|  |  | **2021-2030** | | **2031-2040** | | **2041-2050** | |
| **Category** | **Subcategory** | **MIN** | **MAX** | **MIN** | **MAX** | **MIN** | **MAX** |
| Capital costs | 1. Electric Transport | 0.2024 | 1.0000 | 0.2024 | 1.0000 | 0.2024 | 1.0000 |
|  | 2. ICE Transport | 0.2476 | 1.0000 | 0.2475 | 1.0000 | 0.2469 | 1.0000 |
|  | 3. Industry | 0.1417 | 1.0000 | 0.1417 | 1.0000 | 0.0000 | 0.7798 |
| Fixed costs | 4. Crops | 0.0000 | 0.4994 | 0.0000 | 0.5309 | 0.0000 | 0.4994 |
|  | 5. Electric Transport | 0.0000 | 0.9170 |  |  |  |  |
|  | 6. ICE Transport |  |  |  |  |  |  |
| Variable costs | 7. Crops |  |  |  |  |  |  |
|  | 8. Import Fuels |  |  |  |  |  |  |
| Demand | 9. Fuel Demand |  |  | 0.0000 | 0.3075 |  |  |
|  | 10. Residential Demand |  |  |  |  |  |  |

From the capital costs drivers, the table above shows that mid to higher electric transport capital costs (0.20-1.00) are consistently associated with low emissions across all three periods (2021-2030, 2031-2040, and 2041-2050). This persistent pattern suggests that the cost structure of electric transport remains a relevant driver of low emissions throughout the entire analysis horizon. Similarly, ICE transport capital costs in the mid-to-high range (0.25-1.00) correlate with low emissions in all periods, indicating that more expensive ICE vehicles (slightly more so than electric transport vehicles) may discourage their adoption and indirectly support electrification pathways.

Industry capital costs show an evolving pattern: in the first and second periods (2021-2030 and 2031-2040), the acceptable range for low emissions is relatively broad (0.14-1.00), suggesting that industry capital cost uncertainty has less restrictive influence initially. However, in the third period (2041-2050), this range shifts to 0.0-0.78, indicating that achieving low emissions in the final period becomes more sensitive to keeping industry capital costs within lower-to-mid ranges. This temporal evolution suggests that as the decarbonization pathway matures, the cost structure of industrial transformation becomes increasingly critical.

The analysis identifies that fixed costs associated with crops must remain in the lower half of their range throughout the entire 2021-2050 horizon (0.0-0.50 in the first and third periods, 0.0-0.53 in the second). This consistent constraint across all three periods highlights the importance of agricultural sector cost management as a foundational element of the low-emissions pathway.

Electric transport fixed costs show temporal variation: they are constrained to remain below 0.92 in the nominal range during the first period (2021-2030), but do not emerge as significant drivers in the subsequent periods (2031-2040 and 2041-2050). This suggests that in the first stage fixed cost structures for electric transport infrastructure are critical for establishing the foundation of a low-emission system, but as the transition progresses, other factors become more determinative.

Notably, variable costs have not been identified by PRIM as significant influences on the low-emissions desirable outcome, and demand-side drivers play only a limited role: fuel demand appears solely in the second period (2031-2040), where it must remain in the lower third of its range (0.0-0.31). The near absence of these drivers from the PRIM boxes indicates that, within the explored uncertainty space and PRIM configuration, variations in variable operating costs and demand levels do not systematically differentiate between futures that achieve low emissions and those that do not. This finding suggests that structural factors (capital and fixed costs) are more critical than operational expenses or demand fluctuations for determining emission outcomes in Uganda's context, though these variables may still play important roles in other metrics such as costs or system affordability.

In summary, because the identified driver ranges must be met concurrently within each period, policymakers should interpret these results as integrated conditions rather than independent levers. For example, current results suggest that to achieve low emissions in 2031-2040, Uganda would need to simultaneously ensure that: (1) electric transport capital costs fall within the 0.20-1.00 range, (2) ICE transport capital costs remain in the 0.25-1.00 range, (3) industry capital costs stay between 0.14-1.00, (4) crop fixed costs remain below 0.53, and (5) fuel demand stays below 0.31 of its explored range.

Table 3: Drivers of the risk outcome for Emissions (high)

| **Drivers** | | **Risk outcomes** | | | | | |
| --- | --- | --- | --- | --- | --- | --- | --- |
|  |  | **2021-2030** | | **2031-2040** | | **2041-2050** | |
| **Category** | **Subcategory** | **MIN** | **MAX** | **MIN** | **MAX** | **MIN** | **MAX** |
| Capital costs | 1. Electric Transport | 0.0000 | 0.8863 | 0.0000 | 0.5702 | 0.0000 | 0.5702 |
|  | 2. ICE Transport | 0.0000 | 0.5065 | 0.0000 | 0.7494 | 0.0000 | 0.5056 |
|  | 3. Industry | 0.2674 | 1.0000 | 0.0000 | 0.6870 | 0.2674 | 1.0000 |
| Fixed costs | 4. Crops | 0.0422 | 1.0000 |  |  |  |  |
|  | 5. Electric Transport |  |  | 0.2461 | 1.0000 |  |  |
|  | 6. ICE Transport |  |  |  |  |  |  |
| Variable costs | 7. Crops |  |  |  |  |  |  |
|  | 8. Import Fuels |  |  | 0.0121 | 1.0000 | 0.0121 | 1.0000 |
| Demand | 9. Fuel Demand | 0.0000 | 1.0000 | 0.0000 | 1.0000 | 0.1389 | 1.0000 |
|  | 10. Residential Demand |  |  |  |  |  |  |

The emissions risk analysis reveals that several of the same structural drivers that enable low emissions also delimit the high-emission space, but from the opposite direction. Capital costs appear as significant drivers in all three periods: high-emission futures are characterized by electric transport capital costs in the low-to-mid range (0.0-0.89 in 2021-2030, tightening to 0.0-0.57 in the later periods) and ICE transport capital costs in the lower portion of their range (0.0-0.51 in 2021-2030, 0.0-0.75 in 2031-2040 and 0.0-0.51 in 2041-2050). Industry capital costs above 0.27 accompany high emissions in the first and final periods, while in 2031-2040 the constraint inverts to the lower portion of the range (0.0-0.69), reflecting the non-linear, context-dependent behavior discussed above. Fixed costs play a more secondary role: crop fixed costs above 0.04 appear only in the first period, and electric transport fixed costs above 0.25 only in the second.

Fuel demand emerges as a persistent driver of high-emission risk across all three periods. In the first two decades it spans essentially its full range, meaning that high-emission futures are not distinguished by any particular demand level; by 2041-2050, however, the lower bound rises to 0.14, indicating that even moderately low fuel demand can accompany high-emission outcomes. Several circumstances could contribute to this pattern: cumulative infrastructure lock-in favoring fossil fuels, path dependencies in transportation and energy systems, or the compounding effects of sustained fuel demand on long-term emission trajectories.

Import fuel variable costs appear in the second and third periods (2031-2040 and 2041-2050), essentially spanning nearly the entire uncertainty range (0.01-1.00). Several dynamics could contribute to this correlation: continued fuel consumption despite higher costs (suggesting inelastic demand or lack of viable alternatives), global market conditions where both prices and consumption remain elevated, or scenarios where high fuel costs coincide with heavy fossil fuel dependence without successful energy transition.

### 2.2. Costs

The following two tables show the results obtained for the cost metric.

Table 4: Drivers of the desirable outcome for Costs

| **Drivers** | | **Desirable outcomes** | | | | | |
| --- | --- | --- | --- | --- | --- | --- | --- |
|  |  | **2021-2030** | | **2031-2040** | | **2041-2050** | |
| **Category** | **Subcategory** | **MIN** | **MAX** | **MIN** | **MAX** | **MIN** | **MAX** |
| Capital costs | 1. Electric Transport | 0.0000 | 0.8863 | 0.0000 | 0.8863 | 0.0000 | 0.8863 |
|  | 2. ICE Transport | 0.0000 | 0.6281 | 0.0000 | 0.7494 | 0.0000 | 0.6901 |
|  | 3. Industry | 0.0000 | 0.7763 | 0.0000 | 0.7018 | 0.0000 | 0.7029 |
| Fixed costs | 4. Crops |  |  |  |  |  |  |
|  | 5. Electric Transport |  |  |  |  |  |  |
|  | 6. ICE Transport |  |  | 0.0000 | 0.5072 | 0.0000 | 0.5070 |
| Variable costs | 7. Crops |  |  |  |  |  |  |
|  | 8. Import Fuels |  |  | 0.0000 | 0.5250 |  |  |
| Demand | 9. Fuel Demand | 0.0000 | 0.0000 | 0.0000 | 0.3075 | 0.0000 | 0.3417 |
|  | 10. Residential Demand |  |  |  |  |  |  |

The above pattern indicates that futures achieving desirable (low) cost outcomes in the first decade are characterized by a specific combination where:

- Electric Transport capital costs can range widely (from minimum to 89% of maximum), providing flexibility in EV deployment costs
- ICE Transport capital costs remain in the lower-to-mid portion of their range (up to 63% of maximum), AND
- Industry capital costs also span a broad range (up to 78% of maximum)

In addition, fuel demand is pinned at the very bottom of its explored range (0.00-0.00), indicating that the low-cost futures identified in this decade coincide with minimal fuel demand. The absence of fixed and variable cost drivers suggests capital investment patterns and demand conditions are more influential in the first decade.

For the middle decade (2031-2040), desirable cost outcomes are associated with six concurrent conditions: Electric Transport capital costs up to 89% of maximum, ICE Transport capital costs up to 75%, Industry capital costs up to 70%, ICE Transport fixed costs up to 51%, Import fuel variable costs up to 53%, and Fuel Demand up to 31% of its range. By the final period, desirable cost outcomes are associated with a similar set of five concurrent conditions involving:

- Electric Transport capital costs maintain high flexibility (up to 89% of maximum range), but this occurs simultaneously with:
  - ICE Transport capital costs constrained to ~69% of their maximum
  - Industry capital costs up to 70% of maximum
  - ICE Transport fixed costs up to 51% of maximum
  - Fuel Demand up to 34% of maximum

Some plausible interpretations for the last period are the following

- The persistence of ICE Transport fixed costs from 2031 onwards suggests that operational cost patterns become relevant for differentiating desirable outcomes: keeping the running costs of the legacy fleet in the lower half of their range accompanies low-cost futures.
- The stability of the Industry capital cost condition (constrained to the lower ~70% of its range in all three periods) indicates that moderate industrial investment costs are a consistent precondition for system-wide cost desirability.
- The progressive relaxation of the Fuel Demand condition (0.00 in 2021-2030, up to 31% in 2031-2040 and up to 34% in 2041-2050) suggests that, as the system decarbonizes, low-cost futures become compatible with moderate levels of fuel consumption, occurring alongside the other four conditions.

Table 5: Drivers of the risk outcomes for Costs

| **Drivers** | | **Risk outcomes** | | | | | |
| --- | --- | --- | --- | --- | --- | --- | --- |
|  |  | **2021-2030** | | **2031-2040** | | **2041-2050** | |
| **Category** | **Subcategory** | **MIN** | **MAX** | **MIN** | **MAX** | **MIN** | **MAX** |
| Capital costs | 1. Electric Transport | 0.1049 | 1.0000 | 0.2024 | 1.0000 | 0.0420 | 1.0000 |
|  | 2. ICE Transport | 0.1982 | 1.0000 |  |  |  |  |
|  | 3. Industry | 0.1417 | 1.0000 | 0.1417 | 1.0000 | 0.1417 | 1.0000 |
| Fixed costs | 4. Crops | 0.0000 | 0.5520 | 0.0000 | 0.4994 | 0.0000 | 0.5178 |
|  | 5. Electric Transport | 0.0000 | 0.9170 | 0.0000 | 0.9170 | 0.0000 | 0.9170 |
|  | 6. ICE Transport | 0.0656 | 1.0000 | 0.0654 | 1.0000 | 0.1233 | 1.0000 |
| Variable costs | 7. Crops | 0.2952 | 1.0000 | 0.2952 | 1.0000 | 0.2485 | 1.0000 |
|  | 8. Import Fuels |  |  |  |  |  |  |
| Demand | 9. Fuel Demand |  |  |  |  | 0.0000 | 1.0000 |
|  | 10. Residential Demand |  |  |  |  |  |  |

Finally, undesirable outcomes for costs in the first decade (2021-2030) are associated with futures exhibiting simultaneous variability across seven dimensions, with most allowing very broad ranges:

- Four out of 7 drivers span 86-93% of their full ranges (Electric Transport capital, Industry capital, Electric Transport fixed, ICE Transport fixed), indicating that high-cost outcomes correlate with scenarios where these factors can take on nearly any value within the explored space.
- ICE Transport capital and Crops variable costs notably exclude the lowest portions of their ranges (starting at 0.1982 and 0.2952 respectively), suggesting that very low values in these areas are absent from risk scenarios
- The presence of both capital and operational costs (fixed and variable, including the agricultural sector) from the start suggests that investment patterns AND operating cost structures together influence early-period cost risk.

For the middle decade (2031-2040), the cost-risk profile closely mirrors the first one: six of the same drivers reappear, though only four of them with essentially identical ranges (Industry capital above 0.14, ICE Transport fixed costs above 0.07, crops variable costs above 0.30 and Electric Transport fixed costs up to 0.92). The remaining two tighten: the Electric Transport capital lower bound rises from 0.10 to 0.20 (narrowing the range from 90% to 80% of the full span) and the crop fixed cost ceiling falls from 0.55 to 0.50. ICE Transport capital costs drop out as a significant dimension.

As for the last decade:

- The persistence of Industry capital costs (0.14-1.00) and Electric Transport fixed costs (0.0-0.92) with identical ranges across all three periods suggests these factors maintain consistent influence on cost risk throughout the analysis horizon
- The continued broad ranges for most drivers (75-100%, with only crop fixed costs more constrained at ~52%) suggest that late-period risk still correlates with multi-sectoral variability, though the specific vulnerable dimensions shift: ICE Transport capital costs drop out after the first decade, while Fuel Demand enters in 2041-2050 spanning its full range.

### 2.3. Combining metrics to promote desirable outcomes and avoid risk

To facilitate interpretation, results were organized into two complementary perspectives, presented in the two columns of Table 6. The first identifies co-beneficial conditions (parameter ranges that appear consistently across the desirable cost and desirable emissions matrices), representing low-cost, low-emission futures. The second summarizes trade-offs and vulnerabilities (conditions that appear in the risk matrices or that statistically coincide with cost- or emission-intensive outcomes).

Table 6: Integrated analysis

|  | **Concurrent objectives (low emissions and costs)** | **Caveats (Trade-offs & vulnerabilities)** |
| --- | --- | --- |
| **2021-2030** | Prioritize Electric Transport capital investment: keep at minimum 0.20 to enable transition, below 0.89 for desirable costs outcome. | Capital investment above 10% in EV transport is correlated to high-cost risk when, concurrently: capital investment for ICE transport is above 20%, industry investment is above 14%, ICE transport fixed costs are above 7%, electric transport fixed costs are between minimum and 92% of full range, crops variable costs are above 30% and fixed crop costs are below 55%. |
|  | Constrain ICE Transport capital to the 0.25-1.00 range. Staying below 63% is better for costs. | Capital investment above 20% (incl. the 0.25-0.63 window) might still generate high-cost outcomes if it concurs with the remaining risk conditions: EV transport capital above 10%, industry investment above 14%, ICE fixed costs above 7%, crops variable costs above 30% and fixed crop costs below 55%. |
|  | Invest moderately in the industrial transition: 0.14 to 0.78 of its full range. | Industry investments above 14% of full range also correlate to high-cost outcomes, and above 27% to high-emission outcomes. |
|  | Invest moderately in O&M for Electric Transport: a 0-0.92 range for desirable emissions suggests flexibility; no cost-side constraint was identified for this driver in this period. Note on Fuel Demand: the desirable-costs futures identified in this decade concentrate at the very bottom of the fuel demand range (0.00-0.00). | Fixed costs between 0-92% might still generate high-cost outcomes if: capital investment in EV transport is above 10%, capital investment for ICE transport is above 20%, industry investment is above 14%, ICE fixed costs are above 7%, crops variable costs are above 30% and fixed crop costs are below 55%. High-emission futures span the full fuel demand range (0-100%) in this period, so demand levels alone do not differentiate emission outcomes. |

This dual presentation highlights both reinforcing patterns and exceptions, providing a balanced view of opportunities and risks. The approach does not merge PRIM data quantitatively but cross-references consistent parameter ranges across outcome types^[4]^.

*Table 6 Continuation*

|  | **Concurrent objectives (low emissions and costs)** | **Caveats (Trade-offs & vulnerabilities)** |
| --- | --- | --- |
| **2031-2040** | Continuing Electric Transport momentum (maintain minimum 0.20; staying below 0.89 remains better for costs) | Electric Transport capital investment up to 57% of its range correlates to high-emission risk when, concurrently: ICE Transport capital investment is low to mid-high (0-75%), industry investment is below 69%, electric transport fixed costs are above 25%, import fuel variable costs are above 1% and fuel demand spans its full range (0-100%). Investment above 20% also appears in the high-cost box together with the other cost-risk conditions. |
|  | Moderate ICE Transport: 0.25-1.00 for emissions; staying below 75% is better for costs. | ICE Transport capital investment in the 0-75% range correlates to high-emission risk when, concurrently: Electric Transport capital investment is up to 57%, industry investment is below 69%, electric transport fixed costs are above 25%, import fuel variable costs are above 1% and fuel demand spans its full range (0-100%). |
|  | Industry investment should stay moderate: the 0.14-0.70 window satisfies both the low-emission (0.14-1.00) and low-cost (0.00-0.70) conditions. | Industry investment above 14% of full range also correlates to high-cost outcomes, while values below 69% appear in the high-emission box for this period. |
|  | Manage Fuel Demand: below 31% of its range for both desirable emissions and desirable costs. | Fuel demand spans its full range (0-100%) in the high-emission box for this period, so low demand alone does not prevent emission risk if the other risk conditions concur. |
| **2041-2050** | Sustain Electric Transport investment (minimum 0.20 consistently required for low emissions, no more than 89% of maximum to achieve desirable costs) | Electric transport investment from minimum to 57% also correlates to high-emission risk when: capital investment for ICE transport is around 0-51%, capital investment for industry is above 27%, import fuel variable costs are above 1% and fuel demand is above 14%. Therefore, an electric transport investment between 57% and 89% might correspond to a strategy more aligned to desirable outcomes. |
|  | Maintain low-to-mid ICE Transport capital investments: 0.25-0.69 satisfies both desirable emissions (0.25-1.00) and costs (0.00-0.69). | ICE transport investment from minimum to 51% also correlates to high-emission risk when, concurrently: electric transport investment is up to 57%, capital investment for industry is above 27%, import fuel variable costs are above 1% and fuel demand is above 14%. |
|  | Stricter Fuel Demand limits: maximum 0.34 for desirable costs, and no specific driver range for desirable emissions. | While desirable emission outcomes remain more flexible to fuel demand levels, the risk matrix points to high-emission outcomes when fuel demand is above 14%. The risk is present if that 14% occurs concurrently with: 0-57% electric transport investment, 0-51% capital investment for ICE transport, at least 27% capital investment for industry, and import fuel variable costs above 1%. High-cost futures, in turn, show fuel demand across its whole range. |
|  | Keep Industry capital investment below 70% of its range: the 0.00-0.70 window satisfies both the low-emission (0.00-0.78) and low-cost (0.00-0.70) conditions. | Industry investment above 27% appears in high-emission futures and above 14% in high-cost futures. In addition, crops variable costs above 25% of full range correlate to high-cost risk in this period. |

As the table above shows, when the PRIM matrices are analyzed together, they reveal important tensions for investment strategies. For instance, while electric transport consistently appears as a critical driver of desirable outcomes, the thresholds that minimize emission do not always fully align with those that minimize costs, producing clear trade-offs and internal contradictions across the cost matrices.

## 3. References

Friedman, J. H., & Fisher, N. I. (1999). Bump hunting in high-dimensional data. *Statistics and Computing*, *9*(2), 123–143. https://doi.org/10.1023/A:1008894516817

Howells, R., Strachan, N., Heaps, C., Huntington, H., Kypreos, S., Hughes, A., Silveira, S., DeCarolis, J., & Roehrl, A. (2018). *OSeMOSYS documentation* [Computer software]. https://osemosys.readthedocs.io/en/latest/index.html

Marchau, V., Walker, W. E., Bloemen, P. J. T. M., & Popper, S. W. (Eds.). (2019). *Decision Making under Deep Uncertainty: From Theory to Practice*. Springer International Publishing. https://doi.org/10.1007/978-3-030-05252-2

1. *The desired outcomes, as defined by the current study, are the economic and environmental objectives: low expenditure costs and low GHG emissions.*
2. *Each one of the two conditions of interest is a combination of metrics (costs and emissions) with distinct values (high/low).*
3. *There is an inherent trade-off between coverage and density: as the algorithm peels away more data to increase the concentration of cases of interest (higher density), it simultaneously reduces the proportion of those cases captured (lower coverage). The merit score balances this trade-off by combining both metrics.*
4. Given that PRIM results represent distinct subsets of futures, the way results are grouped or visualized can influence which insights are most apparent. Alternative groupings (metric-by-metric, sectoral, or period-based) may highlight different relationships. The chosen format prioritizes interpretability and the identification of robust co-beneficial conditions rather than statistical exhaustiveness.
